# Trace element accumulation behavior, ability, and propensity of *Taraxacum officinale* F.H. Wigg (Dandelion)

**DOI:** 10.1007/s11356-024-32293-2

**Published:** 2024-02-06

**Authors:** Alaaddin Vural

**Affiliations:** Faculty of Engineering, Department of Geological Engineering, Ankara University, Gölbaşı, 06830 Ankara Türkiye

**Keywords:** Hyperaccumulator, Phytoexatraction, Trace elements (TEs), *Taraxacum officinale* F.H. Wigg (Dandelion), Bio-concentration factors, Translocation factors

## Abstract

The study aims to achieve several objectives: determine background element concentrations in *Taraxacum officinale* F.H. Wigg-common dandelion roots, stems, and flowers; clarify *T. officinale*'s ability to accumulate trace elements (TEs) in plant parts; calculate accumulation and translocation factors to identify hyperaccumulator potential; and contribute to practical applications in geochemical prospecting, exploration geochemistry, phytomining, and environmental remediation. To accomplish this, 34 samples of *T. officinale* and associated soil samples were gathered from the plant's habitat along the roadside for trace element (TE) analysis. The elemental composition of both plants and soils underwent analysis using diverse statistical approaches, including factor analysis, ANOVA tests, and descriptive parameters. Plant element accumulation patterns and tendencies across various plant parts were explored through bioconcentration (BCFs) and translocation factors (TFs). Results showed that *T. officinale* accumulates some TEs in its roots, stems, and flowers, with the highest concentrations found in the roots. The factor analysis of plant element contents indicated a grouping tendency among elements. Specifically, Al, Cd, Co, Cr, Cu, Fe, Mn, Ni, Pb, and Zn exhibited a tendency to co-occur, while Ba, Mo, and Sr formed a separate group. Furthermore, in the flowers of the plant, Al, Ba, Co, Cr, Fe, Mn, Ni, and Sr showed a similar grouping, while Cu, Pb, and Zn formed a distinct cluster. ANOVA tests demonstrated significant variations in the accumulation of Al, Ba, Cr, Fe, and Sr among the roots, flowers, and stems of the plant. Additionally, Ni and Pb exhibited noteworthy differences in accumulation between these plant parts. Furthermore, Mn and Zn showed significant discrepancies in their accumulation capacities across all parts of the plant. *T. officinale* was identified as a lead hyperaccumulator by the study, with BCFs and concentrations exceeding 1000 mg kg^−1^. These findings inform strategies for phytoremediation and enhance our understanding of the plant's potential role in exploration studies and environmental management in metal-rich regions.

**Supplementary Information:**

The online version contains supplementary material available at 10.1007/s11356-024-32293-2.

## Introduction

Understanding the capacity of plants to accumulate elements holds great significance for various purposes. Previously, plants were extensively employed in exploration geochemistry studies (Brooks 1983; McInnes et al. 1996). Nowadays, they are widely utilized as bioindicators of heavy metal (HM) and TE pollution caused by industrialization (Dahlgren 2006; Reid 2008; Soylak and Türkoğlu 1999; Vural 2016). The escalating exposure of soil, water, and plants to TE pollution calls for increased research and studies to investigate and mitigate these pollutants (Adriano 2001; Sungur et al. 2020; Vural 2020, 2015a, 2014; Vural et al. 2018). TE contamination in soil, water, and plants can have far-reaching consequences for the environment and human health. These elements can negatively impact soil quality, inhibit plant growth, disrupt microorganism activity, and contaminate natural water sources, ultimately posing health risks (Adriano 2001; Sungur et al. 2020; Vural 2020, 2015a, 2014). Presently, researchers are actively investigating both human-induced and naturally occurring metals and chemicals present in terrestrial and aquatic environments, seeking solutions to address their pollution. Previously, plants were primarily used in exploration geochemistry, but their capacity to uptake elements was initially examined in phytomining studies (Mc Grath 1998). With environmental pollution becoming a global concern, the uptake of elements by plants was recognized as a valuable tool for remediation. Consequently, plant species serve as valuable bioindicators in pollution studies, and their potential for phytoremediation, phytoextraction, and phytostabilization is actively researched (Adriano et al. 1995; Baker 1981; Brooks 1998; Saracoglu et al. 2007; Vural 2015a, 2014; Zupan et al. 2003). The selection of appropriate plants is crucial for exploration and remediation approaches, with over 400 plant species reported to accumulate metals. *Taraxacum officinale* F.H. Wigg-common dandelion is one such studied bioindicator plant (Bini et al. 2000) and is proposed for remediation due to its ability to accumulate TEs in its above-ground tissues (Turuga et al. 2008). This herbaceous perennial plant belongs to the Asteraceae family, widely distributed in anthropic ecosystems and rural areas, indicating its broad ecological adaptability (TÜGGM 2008).

**Fig. 1:**
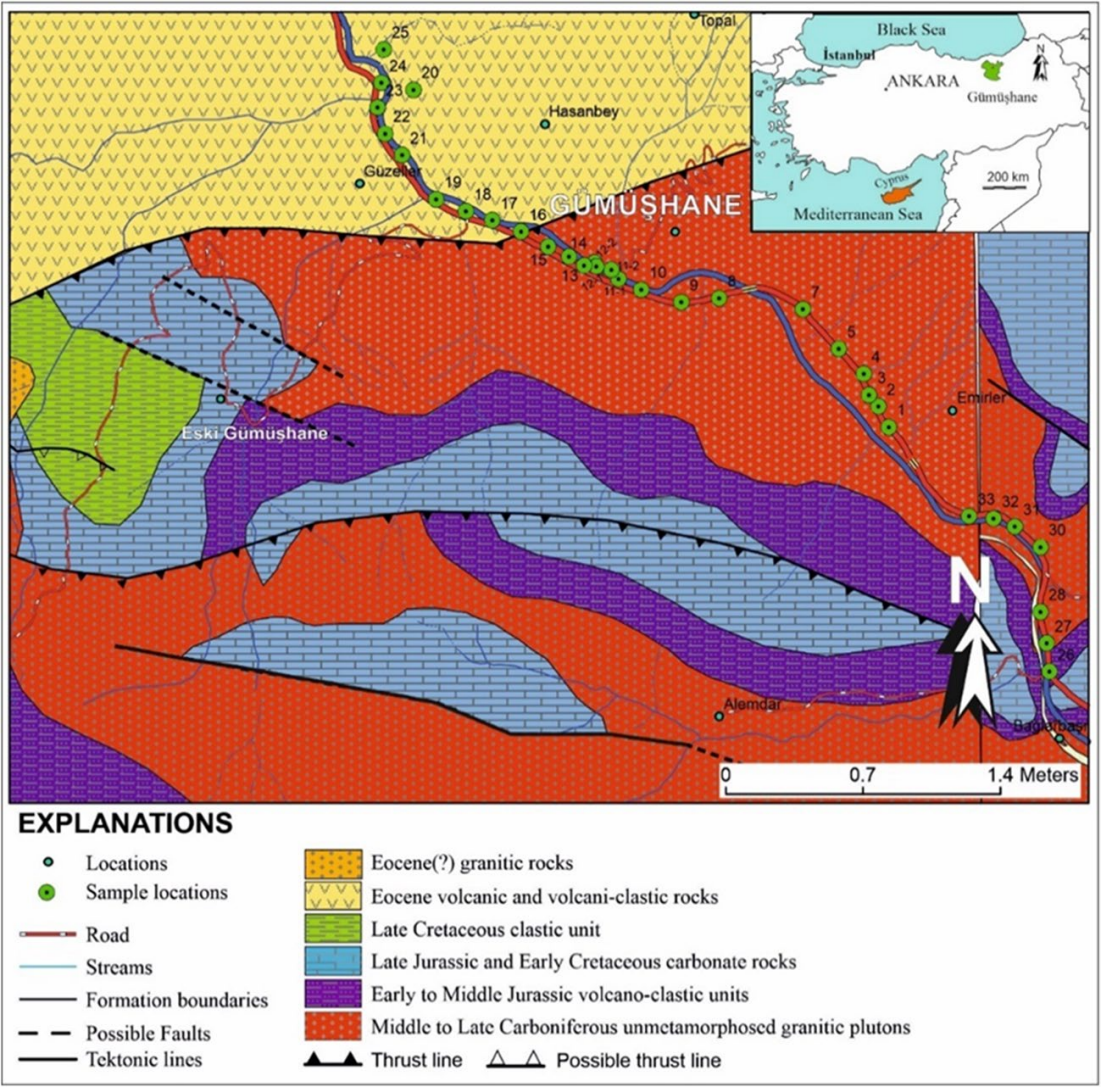
Geological map indicating the distribution of sample locations within the study area (Modified from Güven 1993; Vural 2022)

This study aims to explore the TE uptake and accumulation capacities of *T. officinale* and elucidate its behavior during this phenomenon. *T. officinale* samples were collected from roadside locations adjacent to a highway in Gümüşhane, Türkiye (Fig. 1).

The Eastern Black Sea Tectonic Unit (EBSTU), also known as Pontides, is a part of the Alpine-Himalayan Orogenic Belt. Gümüşhane, located in this unit, is a significant metallogenic belt in Türkiye, housing numerous mineral and ore deposits crucial for the country's economy. Mining activities have been ongoing in the region since ancient times (Vural et al. 2009), with some present operations occurring in historical mining areas (Vural 2019a). The region boasts diverse mineral deposits, including volcano-sedimentary, massive sulfides, skarn, and epithermal deposits, with some being currently mined and others abandoned. Due to the prevalent mining sites, the region exhibits elevated background values of HMs and TEs in the soil, water, and plants, making it a significant natural source of HM and TE pollution risk. *T. officinale* is a common plant found in many areas in Gümüşhane (Türkiye) and its surroundings. Therefore, the element accumulation capacity and element accumulation characteristics of this plant will contribute to both phytoremediation of areas exposed to trace and heavy metal pollution and exploration studies in the search for new mine fields in this region with mining potential. Taking into account all these data, it was decided to investigate the biogeochemical properties of *T. officinale* plants grown in Gümüşhane in this study.

Vural (2014) previously conducted an initial study to assess HM contamination in soils along the Gümüşhane road, revealing severe contamination, particularly of Zn and Pb, with minor Cu contamination. During the aforementioned study, T. officnale samples were also collected from the study area. Therefore, the soil element content data from the aforementioned study were used in this study. As *T. officinale* is a well-established bioindicator for HMs, this study aims to investigate its TE accumulation capacity, behavior, and tendencies in the contaminated area. The expected outcomes of this research are as follows:

1. Determination of the background TE concentrations in *T. officinale* roots, stems, and flowers using advanced statistical analysis.
2. Clarification of *T. officinale*'s ability, propensity, and behavior to accumulate TEs in various plant parts.
3. Calculation of stem accumulation factor (SAF), root accumulation factor (RAF), flower accumulation factor (FAF), stem translocation factor (TFs), and flower translocation factor (TFf), known as bioconcentration and translocation factors. These parameters will help identify elements with hyperaccumulator potential and unveil the potential of phytoextraction.
4. Contribution to the practical applicability of *T. officinale* in geochemical prospecting, exploration geochemistry, phytomining, and environmental issues, based on the obtained findings.

## Material and method

### Geomorphological and geological features of the area

The region's basement rocks consist of Early-Middle Carboniferous metamorphic rocks, primarily found in the south-southeast of Gümüşhane beyond the study area. Within the study area, Middle-Late Carboniferous granites intrude the metamorphic rocks (Vural and Kaygusuz 2019; Yılmaz 1972), covering approximately 50% of the study area. These granites are overlain by the Zimonköy Formation, a volcano-clastic unit of Early to Middle Jurassic age (Eren 1983), which contributes to soil development at some sampling points (Saydam Eker and Arı, 2020). The Berdiga Formation, consisting of late Jurassic—early Cretaceous carbonate rocks (Pelin 1977), overlays the Zimonköy Formation in the study area and its surroundings, indirectly contributing to soil development. The Kermutdere Formation, composed of Late Cretaceous clastic rocks, does not crop out at the sampling points and does not directly contribute to the soils in the sampling location. Eocene volcanic and volcani-clastic rocks overlie these units with an angular unconformity and were intruded by calc-alkaline granitoids of similar age (Vural et al. 2021; Vural and Kaygusuz 2021). The youngest units in the area are Quaternary alluvium and travertine (Vural 2019b; Vural and Külekçi, 2021) (Fig. 1).

### Sampling and analysis

In April and May 2012, thirty-four wild *T. officinale* samples were collected along the Türkiye-Iranian international highway in Gümüşhane during a concurrent soil geochemistry study (Vural 2014). To ensure sufficient sample volume, plants from 3–4 individuals were combined into a single sample per site. The soil sample preparation process is described in Vural (2014). The preparation of *T. officinale* samples involved dividing them into root, stem, and flower parts, followed by washing with tap water and ultrapure water. The materials were then dried (at 50–60 °C for 24 h), ground into fine powder, and sent to the Laboratories of Trabzon Provincial Directorate of Agriculture (Türkiye) for analysis. In the laboratory, the plant samples underwent digestion in a high-pressure and closed-vessel microwave oven (Milestone ETHOS D, Sorisole, Italy) using a 5:1 mixture of hydrogen peroxide (30%) and nitric acid (65%) (Merck Company, Darmstadt, Germany). After the digestion procedure, the clear solutions were diluted and analyzed by ICP-OES (Varian, Inc.Vista-PRO, UK) for the presence of Al, As, Ba, Cd, Co, Cr, Cu, Fe, Mn, Mo, Ni, Pb, Se, Sn, Sr, and Zn. The LOD and RSD values were calculated based on standard deviation measurements from blank solutions and low concentration metal solutions. The accuracy of the measurement system was confirmed by analyzing a certified reference material, showing no significant difference between certified and measured values for all metals (Supplement Table S1).

### Statistical methodology

Descriptive statistical analyses were conducted to assess the element concentrations in different parts of *T.officinale*.The data's normality was evaluated using graphical and statistical methods, including the Kolmogorov–Smirnov and Shapiro–Wilk tests. Correlation analysis was performed to measure the strength and direction of linear relationships between elements in plant parts. The Spearman correlation coefficient was used due to the non-normal distribution of some data.

Factor analysis, specifically Principal Component Analysis (PCA), was employed to identify groups of elements with similar behavior and reduce the number of variables. The Kaiser–Meyer–Olkin and Bartlett Sphericity tests were used to assess the suitability of the data for factor analysis. One-way ANOVA and post hoc analyses were used to compare element accumulation in the flower, root, and stem parts of *T. officinale*.

### Metal accumulation efficiency factors (MAEFs)

To assess metal accumulation efficiency in plants, the study determined the stem accumulation factor (SAF), root accumulation factor (RAF), and flower accumulation factor (FAF), which are also known as bioaccumulation factors (BAFs) or bioconcentration factors (BCFs). Additionally, the stem translocation factor (TFs) and flower translocation factor (TFf) were calculated to measure metal transport from roots to stems and flowers.

The stem accumulation factor (SAF) is the ratio of metal concentration in stems to that in soil (Eq. 1), while the root accumulation factor (RAF) is the ratio of metal concentration in roots to that in soil (Eq. 2). Additionally, the flower accumulation factor (FAF) is the ratio of element concentration in flowers to that in soil (Eq. 3). These equations were originally introduced by Vyslouzilova et al. (2003). TFs is the ratio of element concentration in the plant stems to that in the root (Eq. 4), and the flower translocation factor (TFf) is the ratio of element concentration in the plant flower to that in the root (Eq. 5). These equations were initially presented by Sun et al. (2008).

To determine if a plant is a hyperaccumulator, both accumulation factors (SAF, RAF, FAF) and translocation factors (TFs, TFf) should be considered. Plants with accumulation factors and translocation factors greater than one can be used for phytoextraction, removing metals from specific plant tissues. Additionally, plants with a bioconcentration factor greater than one and a translocation factor less than one have the potential for phytostabilization, reducing metal mobility in specific plant tissues. A plant is classified as a hyperaccumulator if it has an accumulation factor greater than 1 or a translocation factor greater than 1, and it accumulates at least 1000 mg kg − 1 of copper (Cu), cobalt (Co), chromium (Cr), or lead (Pb), or at least 10,000 mg kg − 1 of iron (Fe), manganese (Mn), or zinc (Zn) in total (Vural 2016). Several researchers (Luoma and Bryan 1979; Vural 2015b, 2014; Wahsha et al. 2012; Yoon et al. 2006) have used bioaccumulation coefficient factor (BCF) and translocation factor (TF) to assess metal accumulation efficiency in plants. The mentioned parameters resemble the bioaccumulation coefficient factor (BCF) and translocation factor (TF). The provided equations are modified versions used to calculate these parameters. BCF represents the ratio of metal concentration in roots to soil (Eq. 6), while TF represents the ratio of metal concentration in stems to roots (Eq. 7). To assess if a plant is a metal hyperaccumulator, both BCF and TF should be considered. If both BCF and TF are greater than one, the plant has potential for phytoextraction, which involves using plants to remove metals from soil. If BCF is greater than one and TF is less than one, the plant has potential for phytostabilization, reducing metal mobility in soil.

1 $$SAF=\frac{{[Metals]}_{stem}}{{[Metals]}_{soil}}$$

2 $$RAF=\frac{{[Metals]}_{root}}{{[Metals]}_{soil}}$$

3 $$FAF=\frac{{[Metals]}_{flower}}{{[Metals]}_{soil}}$$

4 $$TFs=\frac{{[Metals]}_{stem}}{{[Metals]}_{root}}$$

5 $$TFf=\frac{{[Metals]}_{flower}}{{[Metals]}_{root}}$$

6 $$BCF=\frac{{[Metals]}_{roots}}{{[Metals]}_{soil}}$$

7 $$TF=\frac{{[Metals]}_{stems}}{{[Metals]}_{soil}}$$

### Results and discussion

**Fig. 2:**
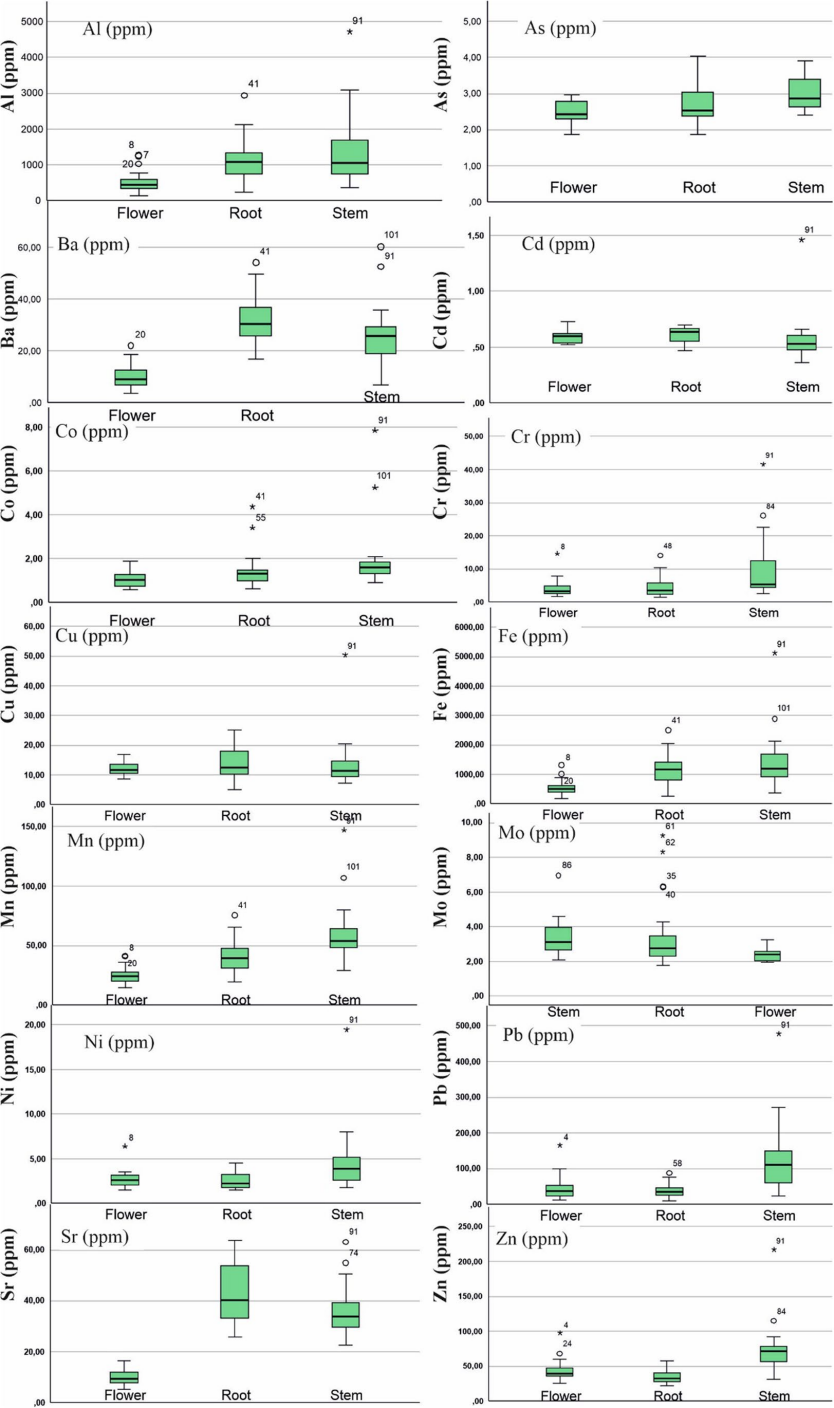
Box plots representing elemental concentrations in the flower, root, and stem of *T. officinale*

**Fig. 3:**
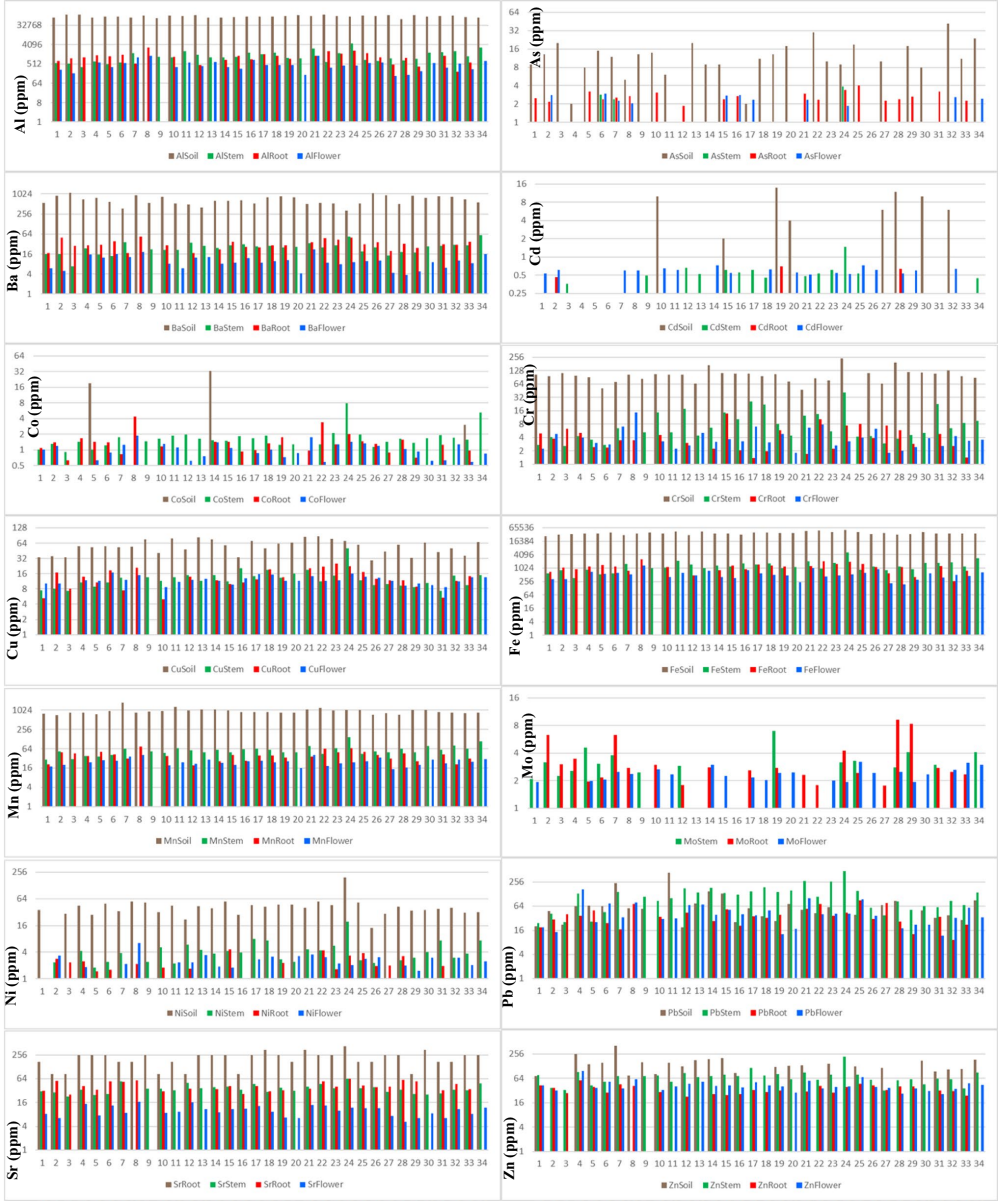
Bar diagram of element concentrations in parts of a *T. officinale* plant and in the soil in which they are grown (The y-axis is on a logarithmic scale with a base of 2)

This study sourced soil element concentrations from Vural (2014). Descriptive statistics of element concentrations in *T. officinale* roots, stems, and flowers were depicted in Fig. 2 and Suplement Table S2, alongside literature values for comparison. Additionally, Fig. 3 presented a bar graph of element contents in soil at sampling points and in the collected *T. officinale* plant parts.

#### Data normality assessment

As some samples of *T. officinale* roots, stems, and flowers had element concentrations below the detection limit, non-parametric tests were used to evaluate the element distribution in these plant components. Both the Kolmogorov–Smirnov and Shapiro–Wilk tests were used to determine whether the distribution of elements followed a normal or logarithmic pattern (Supplement Table S3). In roots, Cd, Co, Cr, Mo, and Ni did not follow a normal distribution, while other elements showed a distribution close to normal (for significance level < 0.05). For stems, only Sr exhibited a distribution close to normal, while the rest did not. In flowers, As, Ba, Cd, Co, Cu, Mn, Mo, and Sr had a distribution close to normal, while the others did not (Supplement Table S3). These results suggest that stems and flowers are more vulnerable to soil pollution. These findings are partially supported by standard deviation, skewness, and kurtosis parameters in descriptive statistics and box plots (Suplement Tables S2, Fig. 2).

Proposed element mean values for plants should be determined based on whether the element concentrations follow a normal distribution or not. If the element concentrations do not exhibit a normal distribution, the median value should be used as the mean value for that element, as the median is less influenced by outliers compared to the arithmetic mean. The arithmetic mean should only be utilized when the element concentrations are normally distributed (Suplement Tables S2 and S3). The average element concentrations (in mg kg − 1) in *T. officinale* roots were as follows: Al: 1128, As: 2.68, Ba: 32.06, Cd: 0.64, Co: 1.32, Cr: 3.61, Cu: 13.66, Fe: 1154.7, Mn: 40.13, Mo: 2.76, Ni: 2.23, Pb: 38.76, Sr: 41.86, Zn: 34.45. In plant stems, the averages were: Al: 1061, As: 2.86, Ba: 25.72, Cd: 0.53, Co: 1.60, Cr: 5.47, Cu: 11.45, Fe: 1184.35, Mn: 53.79, Mo: 3.1, Ni: 3.89, Pb: 109.57, Sr: 33.93, Zn: 71.85. And for plant flowers: Al: 445, As: 2.48, Ba: 9.93, Cd: 0.60, Co: 1.04, Cr: 3.34, Cu: 12.16, Fe: 509.50, Mn: 25.08, Mo: 2.40, Ni: 2.60, Pb: 38.10, Sr: 10.12, Zn: 39.85. Detailed discussions on the element concentrations in roots, stems, and flowers of *T. officinale* are presented below.

#### Aluminum

Aluminum is a common element in plants, typically found in concentrations ranging from 10 mg kg^−1^ to 100 s mg kg^−1^ in higher plants (Kabata-Pendias 2011). However, no comprehensive study on the elemental contents of *T. officinale* roots and flowers has been reported in the literature. In this study, the average aluminum concentration in *T. officinale* roots was 1128 mg kg^−1^, with a range of 228 to 2937 mg kg^−1^. For *T. officinale* stems, aluminum concentrations ranged from 373.13 to 4713.61 mg kg^−1^, with an average value of 1061 mg kg − 1. These findings suggest that *T. officinale* can accumulate high levels of aluminum, which may have implications for human health and the environment. In another study by Djingova and Kuleff (1999), aluminum concentrations in *T. officinale* stems ranged from 65 to 2612 mg kg − 1 (Supplement Table S2), exceeding the values found in some sampling points in the current study. The average aluminum concentration in *T. officinale* flowers was 445 mg kg^−1^ in the present study. Overall, the results indicate that aluminum tends to accumulate more in *T. officinale* stems than in their flowers.

#### Arsenic

Arsenic (As) is present in many plants (ranging from 0.009 to 1.5 mg kg^−1^), and some are known to accumulate it remarkably, making them useful in geochemical prospecting studies (Kabata-Pendias 2011). In our study, As concentrations were detected above the limit in 19 root samples, 3 stem samples, and 11 flower samples of *T. officinale*. The average As concentration in roots was 2.68 mg kg^−1^, ranging from 1.86 to 4.02 mg kg^−1^. For stems, the average was 2.86 mg kg^−1^, ranging from 2.40 to 3.91 mg kg^−1^, and for flowers, it was 2.48 mg kg^−1^, ranging from 1.87 to 2.97 mg kg^−1^. These values are generally higher than the literature reports for *T. officinale*. The As concentration in roots was significantly higher than in stems and flowers (Figs. 2 and 3; Suplement Table S2).

In a study by Vural (2014) in the same study area, acacia trees' shoot samples had an average As concentration of 2.03 mg kg^−1^, which is notably high. This is attributed to the slightly elevated As background values in the sampled soils.

#### Barium

Barium is not essential for plants but is commonly found in them. Plant barium concentrations typically range from 1 to 198 mg kg^−1^ (dry weight, DW), with higher levels in grains and legumes and lower levels in fruits. The toxicity of barium in plants is around 1–2% (Chaudry et al. 1977). In this study, the Ba concentrations were 30.28 mg kg^−1^ in roots, 25.72 mg kg^−1^ in stems, and 8.95 mg kg^−1^ in flowers. These values fall within the expected range for plants, and the roots exhibited higher barium accumulation capacity (Figs. 2 and 3, Suplement Table S2).

#### Cadmium

Cadmium (Cd) is easily absorbed by the roots and stems of plants. Edible plant parts generally contain values below 0.1–0.4 mg kg^−1^ (Kabata-Pendias 2011). In polluted environments, plants may show higher Cd values due to increased absorption. In a study by Bini et al. (2012) from a contaminated area, average Cd values of 0.52 mg kg^−1^ in roots and 0.66 mg kg^−1^ in stems were reported. In the current study area, Cd values above the detection limit were found in 3 root samples, 14 stem samples, and 18 flower samples. The average Cd values in roots, stems, and flowers were 0.64, 0.53, and 0.60 mg kg^−1^, respectively. The roots and flowers of *T. officinale* showed higher Cd accumulation potential. These values approach the upper limits for *T. officinale*, a plant commonly consumed by humans. Hence, when consuming *T. officinale*, considering the collection location is essential (Suplement Table S2, Figs. 2 and 3).

#### Cobalt

Various authors (e.g. Kabata-Pendias 2011) have reported cobalt concentrations in plants to range from 4 to 80 μg/kg. Gworek et al. (2011) found values of 0.39 to 0.61 ppm in roadside-grown *T. officinale* stems, without washing the samples, potentially affected by external factors.

In our study, we assessed cobalt concentrations at nearly all sampling points. The average cobalt concentrations were 1.32 mg kg^−1^ in the roots, 1.6 mg kg^−1^ in the stems, and 1.04 mg kg^−1^ in the flowers. These values significantly exceeded those of Gworek et al. (2011) (Suplement Table S2; Figs. 2 and 3) and were also higher than the values reported for acacia shoots in the same field by Vural (2014). These findings suggest that *T. officinale* might possess a higher ability to accumulate cobalt. Additional investigation is required to corroborate these findings. and explore the underlying mechanisms.

#### Chromium

Chromium levels in plants are typically within the range of 0.02 to 0.2 mg kg^−1^ (Kabata-Pendias 2011). However, in various studies, higher chromium concentrations have been observed in *T. officinale* roots (1.22 mg kg^−1^) and stems (3.16 mg kg^−1^) (Bini et al. 2012). Additionally, in heavily trafficked areas, *T. officinale* plants have shown even higher values, ranging from 3.9 to 4.7 mg kg^−1^ in stems (Gworek et al. 2011). In polluted environments, *T. officinale* stems have exhibited exceptionally elevated levels, reaching up to 61.72 mg kg^−1^ (Keane et al. 2001a).

In this study, chromium concentrations were measured at various sampling points. The average concentrations were 3.61 mg kg − 1 in roots, 5.47 mg kg − 1 in stems, and 3.34 mg kg − 1 in flowers. These values are comparatively lower than those reported in the existing literature. The absence of chromium pollution in the soil samples from the field (Vural 2014) may account for the reduced levels in the plants.

However, when comparing *T. officinale* with other plants, it shows higher chromium concentrations (Suplement Tables S2, Figs. 2 and 3). For instance, wheat grains have a chromium concentration of 0.01 mg kg^−1^, oat grains have 0.09 mg kg^−1^, carrots have an average of 0.13 mg kg^−1^, onions have 0.16 mg kg^−1^, and cabbage contains 0.13 mg kg^−1^. Leafy vegetables and fruits, on the other hand, typically exhibit chromium concentrations between 0.04 and 0.08 mg kg^−1^ (Kabata-Pendias 2011).

#### Copper

Copper is an essential element for both plants and animals. In environments contaminated with copper, plants can accumulate concentrations ranging from 1 to 10 s mg kg^−1^. However, the copper concentration in plant stems generally remains below 20 mg kg^−1^, which is considered the threshold value for plants (Kabata-Pendias 2011).

In the study by Bini et al. (2012), the copper concentration in the roots of *T. officinale* plants was 7 mg kg^−1^ at control points and increased to 49.33 mg kg^−1^ in polluted areas. Similarly, the copper concentration in the stems of *T. officinale* plants was 9 mg kg^−1^ at control points and rose to 52.67 mg kg^−1^ in polluted areas.

In the study by Czarnowska and Milewska (2000), dandelion stems showed an average copper (Cu) concentration of 9.2 mg kg^−1^ at control points and a range of 8.0 to 21.8 mg kg^−1^ at polluted areas. Similar studies by Djingova and Kuleff (1999) and Diatta et al. (2003) reported Cu concentrations in *T. officinale* stems ranging from 4.0 to 22 mg kg^−1^ and 10.1 to 13.9 mg kg^−1^, respectively Suplement Table S2).

In our study, the average Cu concentrations in *T. officinale* roots, stems, and flowers were 13.66 mg kg^−1^, 11.45 mg kg^−1^, and 12.16 mg kg^−1^, respectively. Although Cu concentrations were higher in the roots, they generally remained below the values found in the literature, and the average Cu concentration in the stems did not exceed the recommended 20 mg kg^−1^ threshold for plant stems.

Considering that the soils where *T. officinale* plants grow have copper concentrations above the upper crust values but close to the pollution limit (Vural 2014), higher Cu concentrations in the plants might have been expected. However, the low Cu concentrations found in this study suggest that *T. officinale* may not efficiently accumulate copper (Suplement Table S2; Figs. 2 and 3).

#### Iron

Iron is an essential element for plants, with varying content in forage crops (18 to 1000 mg kg^−1^, DW) and edible vegetables (29 to 130 mg kg^−1^) (Kabata-Pendias 2011). In a study by Bini et al. (2012), the mean iron (Fe) concentrations in *T. officinale* roots and stems were 108 mg kg^−1^ and 470 mg kg^−1^, respectively, in control samples, and 666.67 mg kg^−1^ in polluted environments. Another study by Keane et al. (2001a) found Fe concentrations in *T. officinale* stems of up to 3916 mg kg^−1^.

In our study, the average Fe concentrations in *T. officinale* roots, stems, and flowers were 1154.7 mg kg^−1^, 1184.35 mg kg^−1^, and 509.5 mg kg^−1^, respectively. These values surpassed the average values in Bini et al. (2012)'s control and polluted area samples. Several factors, such as soil type, rainfall, and presence of other metals, may account for the higher Fe concentrations. Further investigation is needed to ascertain the exact reasons behind the elevated Fe levels in this study.

#### Manganese

The concentration of manganese (Mn) in various plants grown in the same soil can range from 30 to 500 mg kg^−1^ (Loneragan 1975). Mn deficiency occurs at levels below 15–25 mg kg^−1^, but toxicity levels are not precisely defined (Kabata-Pendias 2011). In the case of *T. officinale*, there is limited literature on Mn content, with reported average Mn concentrations of 43 mg kg^−1^ in the stems of plants grown in polluted areas (Kabata-Pendias 2011). No evidence of Mn content was found in the roots or flowers.

Our study showed mean Mn concentrations of 40.13 ppm in plant roots, 53.79 ppm in stems, and 25.08 ppm in flowers. The stem exhibited a slightly higher Mn concentration compared to stems from polluted areas, indicating a greater propensity for Mn accumulation in the stem. Considering that the soil Mn values were higher than the average, the plant seems susceptible to Mn pollution (Suplement Table S2; Figs. 2 and 3).

#### Molybdenum

While certain plants can tolerate molybdenum (Mo) concentrations as high as 350 mg kg^−1^ without toxicity, areas with Mo-toxic herbivores have reported Mo content ranging from 1.5 to 5 mg kg^−1^. Feeding farm animals with feeds containing over 10 mg kg^−1^ of Mo can lead to severe issues (Kabata-Pendias 2011). Mo is easily absorbed by plants (due to its mobility), resulting in high concentrations in Mo-contaminated regions.

In a study on pollution at a railway junction by Malawska and Wilkomirski (2001), plant stems showed Mo values between 1.5 and 2.5 mg kg^−1^. In the study area, *T. officinale* roots exhibited Mo concentrations ranging from 1.76 to 9.25 mg kg^−1^ (average: 2.76 mg kg^−1^), stems had 2.08 to 6.96 mg kg^−1^ (average: 3.1 mg kg^−1^), and flowers contained 1.92 to 3.22 mg kg^−1^ (average: 2.4 mg kg^−1^). Some of these values approached toxic levels.

In a study by Vural (2014) on acacia shoots in the same area, the average molybdenum (Mo) concentration was 23.05 mg kg^−1^, ranging from 2.34 to 56.45 mg kg^−1^. Limited literature on *T. officinale* Mo content suggests lower values compared to acacia shoots in the field. However, some *T. officinale* plants, particularly those consumed by herbivorous animals, showed higher Mo values, surpassing the upper limit of 10 mg kg^−1^. As *T. officinale* is commonly consumed by people in various regions, the background Mo values of its growing areas should be taken into consideration (Suplement Table S2; Figs. 2 and 3).

#### Nickel

The nickel (Ni) concentration in forage grasses ranges from 0.1 to 1.7 mg kg^−1^, while in clover, it varies between 1.2 and 2.7 mg kg^−1^. Limited data exist for Ni content in vegetables. Previous studies on *T. officinale* found Ni concentrations of 0.9 mg kg^−1^ in uncontaminated areas and 1.8–3.2 mg kg^−1^ in polluted areas (Czarnowska and Milewska 2000). Other studies on HM pollution reported Ni concentrations in *T. officinale* stems ranging from 2.2 to 2.8 mg kg^−1^, 2.9 to 6.1 mg kg^−1^, and 2.38 to 22.69 mg kg^−1^ (Diatta et al. 2003; Gworek et al. 2011; Keane et al. 2001b).

In our study, Ni concentrations in *T. officinale* roots, stems, and flowers ranged from 1.47 to 4.57 mg kg^−1^, 1.79 to 19.49 mg kg^−1^, and 1.51 to 6.4 mg kg^−1^, respectively. These values were slightly higher than those reported for forage crops, with stems showing significantly higher Ni concentrations than roots and flowers (Suplement Table S2; Figs. 2 and 3). The stem's ability to accumulate Ni from the soil could account for the higher concentrations. While Ni is essential for plants, high levels can be toxic. Further research is needed to understand the impact of Ni on the growth and development of *T. officinale* plants.

#### Lead

Lead is a significant environmental pollutant, and human activities, particularly in industrially developed regions, have notably increased lead levels in plants, fruits, and vegetables. Geological sources also contribute to high lead content in plants (Vural 2015a). Typically, lead content in plants from uncontaminated areas falls within the range of 1.5–2.4 mg kg^−1^ (Alexander et al. 2006; Kabata-Pendias 2011). The lead levels originating from geological environments can reach remarkably high values in plants (Vural 2015a).

Public authorities, such as the Food Additives and Pollution Codex (FAPC), establish lead limits for various plants: grains (0.2 mg kg^−1^), potatoes (0.1 mg kg^−1^, fresh weight, FW), and other vegetables (0.05–0.01 mg kg^−1^, FW) (Kabata-Pendias 2011). These limits are based on the average lead content in plants from uncontaminated areas. However, in regions affected by lead pollution, plant lead levels can significantly exceed these limits.

In a study by Bini et al. (2012), lead concentrations in *T. officinale* roots from uncontaminated areas averaged 1.41 mg kg^−1^, while in contaminated areas, the average was 119.93 mg kg^−1^. Similarly, lead concentrations in stems from uncontaminated areas averaged 3.32 mg kg^−1^, and in contaminated areas, they ranged from 3.32 to 153.33 mg kg^−1^. Keane et al. (2001a, b) also reported comparable findings, indicating lower lead concentrations in plants from uncontaminated areas and higher levels in polluted environments (Suplement Table S2).

In our study, lead concentrations in *T. officinale* roots, stems, and flowers from a contaminated area were 9.33–87.78 mg kg^−1^, 24.28–476.97 mg kg^−1^, and 11.60–165 mg kg^−1^, respectively, with average concentrations of 38.76 mg kg^−1^, 109.57 mg kg^−1^, and 38.10 mg kg^−1^, respectively. These values significantly exceeded those reported in the literature. The highest lead concentration was found in the stems, indicating that plants' lead content is influenced by their growing environment. Therefore, it is essential to investigate the environment before consuming plants to avoid potential harm to human health (Suplement Table S2; Figs. 2 and 3).

#### Strontium

Strontium is an alkaline earth element, mainly found as Sr^2+^ in solution, and does not form strong complexes with organic or inorganic compounds (Lefevre et al. 1993). It has high solubility, limiting dissolved concentrations in soil and groundwater. Strontium is readily taken up by plants, leading to high phytoconcentration, with plant strontium content ranging from 1 to 10,000 mg kg^−1^ (DW) (Kabata-Pendias 2011). Common strontium levels in food and forage plants fall within 10 to 1,500 mg kg^−1^ (DW), with lower values in fruit and edible tubers and higher values (219–662 mg kg^−1^) in legume grasses (Kabata-Pendias 2011).

Research on strontium toxicity is limited, with Shacklette et al. (1978) reporting 30 ppm (atoms per weight, AW) as the toxicity level for plants. Studies have also shown that strontium tends to accumulate more in shoots than in leaves of various plants (Bowen and Dymond 1955). For instance, *Cassia auriculata* contains significant strontium amounts in both leaves and shoots, with 926 and 1,364 mg kg^−1^, respectively (Raghu 2001). For *T. officinale* stems, the strontium content was found to range from 43.5 to 44.9 mg.kg^−1^ (Gworek et al. 2011).

In this study, *T. officinale* plants grown in a limestone-rich area showed strontium concentrations of 25.76–63.75 mg kg^−1^ in roots, 22.49–63.12 mg kg^−1^ in stems, and 5.26–16.50 mg kg^−1^ in flowers. The average strontium concentrations were 41.86 mg kg^−1^ in roots, 33.93 mg kg^−1^ in stems, and 10.12 mg kg^−1^ in flowers. These values were slightly higher than those reported by Shacklette et al. (1978). The elevated strontium concentrations in the plants are likely due to the limestone-rich soils derived from the Berdiga Formation in the study area.

#### Zinc

The zinc content in plants can vary significantly based on factors like plant species, soil type, and growing conditions. Generally, zinc is more concentrated in roots, greens (leaves), shoots, and stems, in that order (Kabata-Pendias 2011). Zinc is considered a mobile element in plants, capable of moving from one part to another, but its mobility is restricted by physicochemical conditions.

Grass and alfalfa plants globally contain zinc concentrations ranging from 12 to 47 mg kg^−1^ and 24 to 45 mg kg^−1^, respectively. For cereal grains like wheat and oat, zinc concentrations vary between 24 and 33 mg kg^−1^ (Kabata-Pendias 2011). US food reference values report zinc content in certain vegetables between 0.7 and 8 mg kg^−1^, fruits between 0.4 and 3 mg kg^−1^, cereals between 0.7 and 32.5 mg kg^−1^, and hazelnut group fruits between 5 and 42.3 mg kg^−1^ (Ensminger et al. 1995; Kabata-Pendias 2011).

Multiple studies have examined zinc concentrations in *T. officinale* plants grown in both clean and polluted areas. Bini et al.. (2012) found that in clean areas, the average zinc content in the roots was 33 mg kg^−1^, whereas in polluted areas, it increased to 65.67 mg kg^−1^. Similarly, in clean areas, the average zinc concentration in the stems was 44 mg kg^−1^, while in polluted areas, it rose to 135 mg kg^−1^. Czarnowska and Milewska (2000) reported an average zinc concentration of 34 mg kg^−1^ in the stems of *T. officinale* plants from a control area. In polluted areas, the zinc content in the stems ranged from 33 to 203 mg kg^−1^, as observed in the study by Diatta et al. (2003), where the range was 27.4 to 86 mg kg^−1^. Studies conducted by Djingova and Kuleff (1999), Gworek et al. (2011), and Keane et al. (2001a) showed that zinc concentrations in *T. officinale* stems from polluted areas ranged from 17 to 90 mg kg^−1^, 45 to 175.6 mg kg^−1^, and 29 to 261.4 mg kg^−1^, respectively (Suplement Table S2). These findings highlight the significant variation in zinc content depending on pollution levels in the growth environment. *T. officinale* plants from polluted areas may contain elevated zinc levels, which can pose potential risks to human health.

In this study, zinc concentrations in *T. officinale* plants were measured in the roots, stems, and flowers, ranging from 22.26 to 57.39 mg kg^−1^, 31.41 to 216.58 mg kg^−1^, and 25.80 to 97.50 mg kg^−1^, respectively. The average zinc concentrations were 34.45 mg kg^−1^, 71.85 mg kg^−1^, and 39.85 mg kg^−1^, respectively. Notably, the stems exhibited the highest average zinc concentration. These values were notably higher than the zinc content of vegetables and fruits in the US food references. For instance, while the mean zinc concentration in spinach was 2.8 mg kg^−1^, in this study, *T. officinale* leaves displayed an average of 39.85 mg kg^−1^. Consequently, the zinc concentrations observed in this study were significantly elevated compared to control samples of *T. officinale* from other studies.

### Elemental ınterrelationships in plant parts

The Spearman correlation coefficients for elements in plant roots, stems, and flowers are presented in Suplement Table S4. In the root analysis, aluminum showed weak positive correlations with arsenic (0.49), moderate positive correlations with barium (0.64), cobalt (0.60), copper (0.57), and lead (0.59), high positive correlation with manganese (0.82), and very high positive correlation with iron (0.94). Aluminum also exhibited a weak negative correlation with cadmium (-0.50). Barium demonstrated moderate positive correlations with cobalt (0.58), copper (0.62), and iron (0.63), as well as a highly positive correlation with manganese (0.75). Cobalt had moderate positive correlations with copper (0.53), iron (0.66), and manganese (0.58), and a weak correlation with nickel. The very high positive correlation of cadmium (1.00) with cobalt, iron, and manganese should be interpreted cautiously, given the limited number of element pairs analyzed (only three samples).

Chromium and nickel exhibited a highly positive correlation (0.86) in the plant root, indicating their likely co-occurrence. Copper and iron showed a moderately positive correlation (0.57) in the root. Iron displayed moderate positive correlations with lead (0.63) and highly positive correlations with manganese (0.83). Manganese showed a moderately positive relationship with lead (0.56). Molybdenum exhibited a weak negative correlation (-0.49) with lead, while nickel showed a weak correlation (0.49) with strontium.

The study investigated the relationships between various elements in plant stems. Aluminum showed moderate positive correlations with copper (0.66), nickel (0.56), lead (0.57), and high positive correlations with barium (0.79), cobalt (0.84), chromium (0.71), iron (0.94), and manganese (0.86). Due to limited data, the relationship of arsenic (As) with other elements was not examined. Barium exhibited moderate positive correlations with cobalt (0.69), copper (0.66), manganese (0.69), nickel (0.67), lead (0.68), and strontium (0.66), and weakly correlated with zinc (0.54). It also showed high positive correlations with chromium (0.74) and iron (0.80). Cadmium displayed moderate positive correlations with chromium (0.55) and strontium (0.57). Cobalt showed moderate positive correlations with chromium (0.61), copper (0.58), nickel (0.62), and lead (0.63), and high positive correlations with iron and manganese (0.79 and 0.74, respectively). Chromium demonstrated moderate positive correlations with copper (0.56), iron (0.69), manganese (0.50), lead (0.62), strontium (0.59), and zinc (0.56). Copper showed moderate positive correlations with iron (0.62), manganese (0.52), and zinc (0.59), and high positive correlation with lead (0.86). Iron was moderately positively correlated with nickel (0.51) and lead (0.56), and highly positively correlated with manganese (0.90). Nickel was moderately positively correlated with lead (0.54) and zinc (0.66). Lead exhibited high positive correlations with strontium (0.71) and zinc (0.73), indicating their likely joint behavior in the plant stem. Strontium and zinc also showed a high positive correlation with each other (0.70).

The study examined element relationships in plant flowers. Aluminum displayed moderate positive correlations with chromium (0.58), copper (0.56), lead (0.51), strontium (0.64), and zinc (0.50), and high positive correlations with barium (0.84) and manganese (0.86). It showed a very high positive correlation with iron (0.93), indicating similar behavior among aluminum, iron, and manganese. Arsenic was detected in limited flowers, hindering its correlation analysis with other elements. Barium exhibited moderate positive correlations with chromium (0.60), copper (0.62), lead (0.60), and zinc (0.60), and high positive correlations with iron (0.81), manganese (0.82), and strontium (0.71), indicating shared behavior with these elements in plant flowers. A high positive correlation coefficient (0.71) was found between cadmium and molybdenum, but this relationship requires confirmation through additional data from other studies due to the limited samples available.

The study observed moderate positive correlations between chromium and strontium (coefficient 0.51). Copper displayed moderate positive correlations with iron (0.63), manganese (0.57), lead (0.68), and strontium (0.65), while a high positive correlation of 0.72 was found between copper and zinc, indicating their joint behavior in plant flowers. Iron showed moderate positive correlations with lead (0.58) and zinc (0.57), and high positive correlations with manganese (0.85) and strontium (0.72). Lead exhibited high positive correlations with strontium (0.80) and zinc (0.82), and a moderate positive correlation of 0.69 was observed between strontium and zinc.

The study also employed factor analysis to examine element relationships in plant parts separately and the entire plant. The feasibility of factor analysis for elements in plant roots was evaluated using the Kaiser–Meyer–Olkin (KMO) measure and Bartlett's test. However, the results indicated that factor analysis would not be suitable for plant roots.

Factor analysis was also conducted on the plant flower data, excluding arsenic (As) elements. The suitability of the analysis was confirmed with a Kaiser–Meyer–Olkin (KMO) measure of sampling adequacy at 52.8% and a significant Bartlett's test (p < 0.001) (Suplement Table S5a-c). Three factors with eigenvalues greater than 1 were identified, explaining 86.23% of the total variance (Suplement Table S5a). Varimax rotation was applied to the factors to minimize factor loadings (Suplement Table S5b). The results grouped elements as follows (Suplement Table S5c):

- Al, Ba, Co, Cr, Fe, Mn, Ni, and Sr in Factor 1 (FF1).
- Cu, Pb, and Zn in Factor 2 (FF2).
- Cd and Mo in Factor 3 (FF3).
  This indicates that elements within each group may be involved in similar biological or biogeochemical processes in plant flowers.

**Fig. 4:**
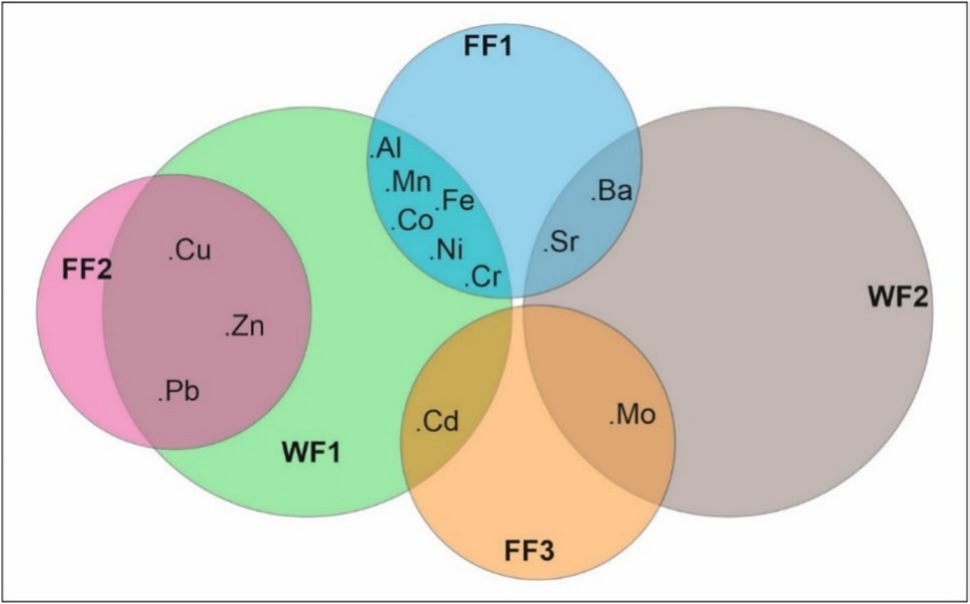
A Venn diagram illustrates the shared factors between a plant's flowers (FFs) and the whole plant (WFs)

In this study, factor analysis was initially attempted to analyze the behavioral tendencies of elements in the plant stem. However, it was found to be unsuitable for the dataset. Subsequently, factor analysis was conducted to identify behavioral groups of elements in *T. officinale* (Suplement Table S5d-f). The suitability of the dataset was confirmed by Kaiser–Meyer–Olkin measure (78.9%) and Bartlett's test (p < 0.000). The analysis revealed two distinct factors/groups, where the first factor explained 67.35% of the variance, and together with the second factor, they accounted for 91.30% of the total variance (Suplement Table S5e-f). The elements in the dataset were grouped accordingly: Al, Cd, Co, Cr, Cu, Fe, Mn, Ni, Pb, and Zn in the first factor, and Ba, Mo, and Sr in the second factor (Suplement Table S5f). The shared factors between the plant's flowers (FFs) and the whole plant (WFs) are depicted in Fig. 4 using a Venn diagram.

Element accumulation capacity and concentration were assessed using the Kruskal–Wallis H test, with Tamhane's T2 post-hoc test applied for further analysis (Suplement Table S6-S7). The choice of Kruskal–Wallis H test over ANOVA was due to the non-normal distribution of elemental data in both the whole plant and its parts. As, Cd, and Cu elements exhibited no significant difference in accumulation capacity, but other elements showed significant differences in at least one plant part compared to others (Suplement Table S5f). Caution is advised when interpreting results for Cd and As due to their low detection limits in some plant parts, resulting in insufficient data. Tamhane's T2 test (Suplement Table S6-S7) revealed significant differences in accumulation capacity for various elements among plant roots, flowers, and stems. Additionally, Ni and Pb elements showed differences in accumulation between roots and stems, as well as between stems and flowers. Mn and Zn elements exhibited varying accumulation abilities across all three plant parts (root, stem, and flower).

### Metal accumulation efficiency in *T. officinale*

**Fig. 5:**
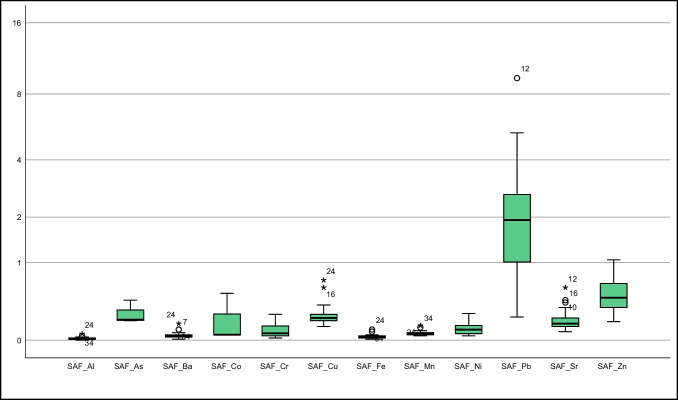
Box plot of SAF (The Y-axis is logarithmically scaled with a base of 2)

The element accumulation characteristics of *T. officinale* parts were analyzed using various parameters such as SAF, RAF, FAF, TFs, and TFf (Suplement Table S8, Fig. 5). In terms of SAF, only the Pb element showed an average value greater than 1 (2.41), with over 75% of Pb SAF values exceeding 1 (Fig. 5). Zn had the second-highest average SAF value of 0.5 among other elements.

**Fig. 6:**
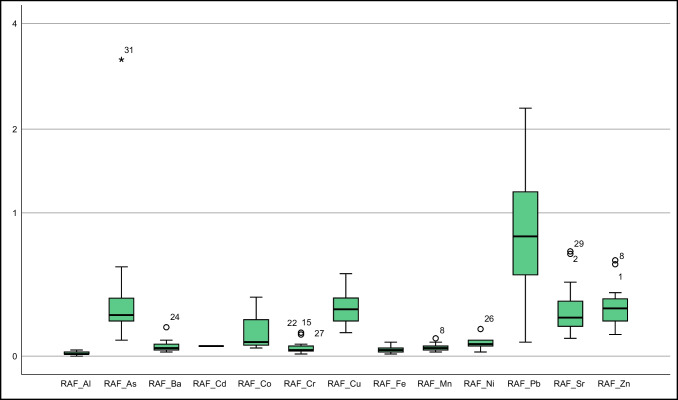
Box plot of RAF (The Y-axis is logarithmically scaled with a base of 2)

Regarding the RAF parameters, no element exhibited average RAF values exceeding 1 (Suplement Table S8, Fig. 6). Pb had the highest RAF value, but more than 50% of its values were below 1 (mean: 0.93, max: 2.32). Arsenic was the only element that exceeded 1 in one sample point among the others.

**Fig. 7:**
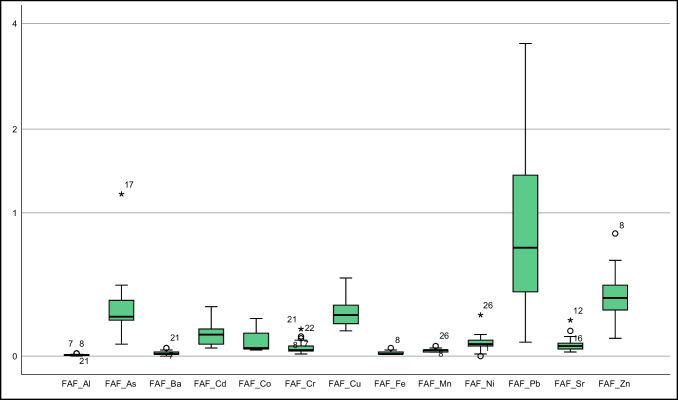
Box plot of FAF (The Y-axis is logarithmically scaled with a base of 2)

The FAF values of *T. officinale* were analyzed, revealing that the average of elements other than Pb was notably below 1, while the average for Pb was approximately 1 (0.99) (Suplement Table S8). Over 50% of the FAF values for lead were below 1, and at least 25% were above 1 (Fig. 7). As the Mo element was not measured in the soil, bioconcentration factors for it couldn't be calculated. Conducting a separate study for *T. officinale* with its soil values is suggested.

**Fig. 8:**
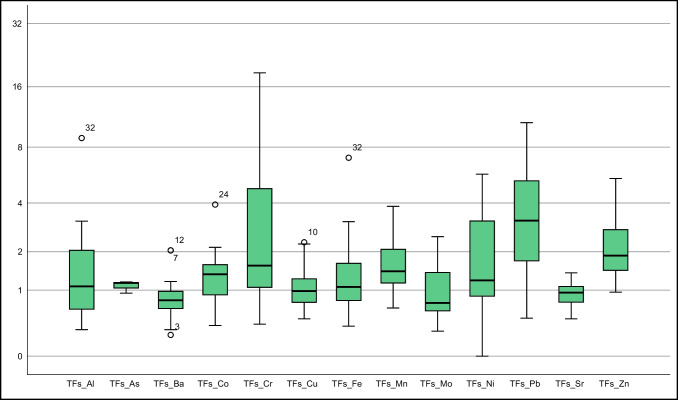
Box plot of TFs (The Y-axis is logarithmically scaled with a base of 2)

Regarding translocation factors (TFs, TFf) of *T. officinale*, TFs averages were found to be greater than 1 for all elements except Sr and Ba (0.86, 0.91, respectively) (Suplement Table S8). This indicates that *T. officinale* has a higher ability to accumulate elements in the stem compared to the root. More than 50% of elements Al, Cu, and Fe, all of As (for 3 samples), almost 70% of Co, over 75% of Cr, almost all of Pb and Mn, more than 25% of Mo, almost 75% of Ni, close to 50% of Sr, and all of Zn had TFs greater than 1 (Fig. 8). The Kruskal–Wallis H and Post Hoc Tamhane's T2 test data suggest significant differences in TFs values for the elements Cr, Mn, Pb, and Zn. Further studies with an expanded data set for other elements nearing the limit are recommended.

**Fig. 9:**
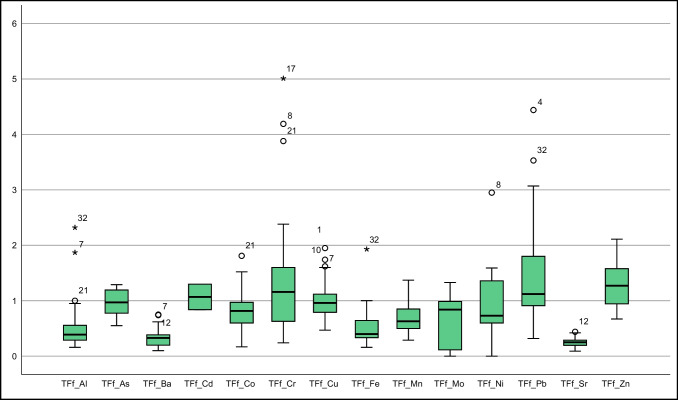
Box plot of TFf (The Y-axis is logarithmically scaled with a base of 2)

The TFf parameters of various elements in *T. officinale* were analyzed. Cd, Cr, Cu, Pb, and Zn had TFf values greater than 1, while Al, Ba, Fe, and Sr had TFf values less than or equal to 0.50. Mn and Mo had TFf values in the range of 0.60–0.70, and As and Ni had TFf values close to 1 (Suplement Table S8, Fig. 8). Notably, approximately 50% of As and Cu samples, over 50% of Cr and Pb samples, and almost 75% of Zn samples had TFf values above 1 (Fig. 9). The Kruskal–Wallis H and Post Hoc Tamhane's T2 test revealed significant differences in TFf values for Al, Ba, Fe, Mn, Sr, and Zn elements. It is essential to note that some elements exhibited a preference for root accumulation.

Considering both bioaccumulation factors (BCFs) and translocation factors (TFs) along with element concentrations in the plant (Suplement Table S9), *T. officinale* was identified as a hyperaccumulator for the Pb element. This conclusion is supported by BCFs for Pb (RAF, SAF, and FAF) being greater than 1, TF being greater than 1, and the concentration of Pb in *T. officinale* exceeding 1000 mg kg^−1^.

## Conclusions

This comprehensive study investigated the trace element (TE) uptake and accumulation capacity of *T. officinale* growing in Gümüşhane, Türkiye. The study area is characterized by elevated TE/metal concentrations due to its geological background and/or anthropogenic activities, such as mining and heavy traffic. The study revealed that *T. officinale* readily accumulates TEs in its roots, stems, and flowers, with the highest concentrations found in the roots.

Several significant findings emerged from the study:

- *T. officinale* as a bioindicator: *T. officinale* effectively accumulates TEs, confirming its suitability as a bioindicator for monitoring TE contamination in the environment.
- Differential element accumulation: Plant parts exhibited distinct TE accumulation patterns, with roots accumulating the highest levels followed by stems and flowers. This suggests the potential for using *T. officinale* in both phytoextraction and phytostabilization strategies depending on the element and plant part targeted.
- Lead hyperaccumulation: *T. officinale* demonstrated hyperaccumulation potential for lead (Pb), exceeding established thresholds for both bioaccumulation and translocation factors.
- Elemental relationships: PCA revealed distinct groups of elements with similar behavior patterns, providing valuable insights into their uptake and translocation mechanisms.
- Co-occurrence and correlations: Significant positive correlations observed between several elements suggest co-occurrence or shared biogeochemical processes, requiring further investigation.

Overall, the study highlights the promising potential of *T. officinale* for both phytoextraction and phytostabilization of TE-rich/contaminated soil. Further studies, including field trials and investigations of various factors influencing TE uptake and accumulation, are necessary to optimize its use for these purposes. Additionally, research on potential health risks associated with consuming *T. officinale* from contaminated environments is crucial.

In conclusion, the study underscores the significance of *T. officinale* as a bioindicator and phytoremediation agent in TE/metal-rich environments. The outcomes not only advance our knowledge of *T. officinale*'s behavior in TE/metal-contaminated soils but also offer practical implications for sustainable resource exploration and pollution mitigation. The integration of diverse analytical approaches provides a robust foundation for future research on plant-metal interactions and environmental management strategies. As environmental concerns continue to grow, this study's insights contribute to the broader discourse on the role of plants in promoting environmental sustainability and human well-being.

### Supplementary Information

Below is the link to the electronic supplementary material.

Supplementary file1 (DOCX 110 KB)

## Data Availability

Data supporting Figs. 5, 6, 7, 8 and 9, Suplement Tables S8 (for the concentrations of elements in the soil) can be accessed at https://dergipark.org.tr/tr/download/article-file/524011 (Vural 2014, Suplement Tables S1). Data supporting Figs. 2, 3 and 4, and Figs. 5, 6, 7, 8 and 9 (for the concentrations of elements in the *T. officinale*’parts), Suplement Table contents (for the concentrations of elements in the *T. officinale*’parts) are avaible on request from the corresponding author.

## Declarations

This article does not contain any studies with human participants or animals performed by the author.
