# Supplementary material for: Trace element accumulation behavior, ability, and propensity of Taraxacum officinale F.H. Wigg (Dandelion)
Source: Environ Sci Pollut Res Int. 2024 Feb 6;31(11):16667–84. doi: 10.1007/s11356-024-32293-2 (PMC10894182; doi:10.1007/s11356-024-32293-2)
Supplement: Supplementary file 1 — Supplementary file1 (DOCX 110 KB) [file 11356_2024_32293_MOESM1_ESM.docx]

**Trace Element accumulation behavior, ability, and propensity of** ***Taraxacum officinale* L. (Dandelion)**

**Supplementary Materials**

Alaaddin Vural^a^*

^a^ Ankara University, Faculty of Engineering, Department of Geological Engineering, Gölbaşı, 06830, Ankara-Türkiye

*Corresponding author: Alaaddin Vural

Address: Ankara University, Faculty of Engineering, Department of Geological Engineering, Gölbaşı, 06830, Ankara-Türkiye

Email: alaaddinvural@hotmail.com

Table S1 Certified reference material analysis as accuracy test for microwave / ICP-AES method (CRM 1568a Rice Flour)

|  | Al | As | Ba | Cd | Co | Cr | Cu | Fe | Mn | Mo | Ni | Pb | Se | Sn | Sr | Zn |
| --- | --- | --- | --- | --- | --- | --- | --- | --- | --- | --- | --- | --- | --- | --- | --- | --- |
| Certified values (mg/kg) | 4.4±1.0 | 0.29±0.03 | * | 0.022±0.002 | 0.018 | * | 2.4±0.3 | 7.4±0.9 | 20.0±1.6 | 1.46±0.08 | * | (<0.010) | 0.38±0.04 | -0.0047 | * | 19.4±0.5 |
| Obtained values (mg/kg) | 4.3±0.5 | BDL | 0.43±0.02 | BDL | BDL | BDL | 2.2±0.2 | 7.6±0.4 | 18.8±0.2 | BDL | BDL | BLD | BDL | BDL | 0.18±0.01 | 17.5±0.5 |
| Error (%) | 2.3 | - | - | - | - | - | 8.3 | 2.7 | 6.0 | - | - | - | - | - | - | 9.8 |

BDL: Below the detection limit

Table S2 Descriptive statistics of root, stem, and flowers of the *T. officinale*

| **Descriptive statistics of root of the *T. officinale*** | | | | | | | |
| --- | --- | --- | --- | --- | --- | --- | --- |
|  | **Mean** | **Median** | **S.D.** | **Min.** | **Max.** | C^1^ | C^2^ |
| **Al** | **1128** | 1088.7 | 580.19 | 228.22 | 2937.6 |  |  |
| **As** | **2.68** | 2.54 | 0.51 | 1.86 | 4.02 |  |  |
| **Ba** | **32.06** | 30.28 | 9.91 | 16.98 | 53.95 |  |  |
| **Cd** | 0.6 | **0.64** | 0.12 | 0.47 | 0.7 | 1.33±0.4 | 0.52 |
| **Co** | 1.45 | **1.32** | 0.8 | 0.63 | 4.37 |  |  |
| **Cr** | 4.5 | **3.61** | 2.95 | 1.4 | 14.12 | BDL | 1.22 |
| **Cu** | **13.66** | 12.51 | 5.5 | 4.96 | 25.18 | 7±1 | 49.33 |
| **Fe** | **1154.7** | 1169.4 | 499.7 | 263.4 | 2505.6 | 108±27 | 224.67 |
| **Mn** | **40.13** | 39.27 | 13.61 | 19.48 | 75.53 |  |  |
| **Mo** | 3.48 | **2.76** | 2.12 | 1.76 | 9.25 |  |  |
| **Ni** | 2.53 | **2.23** | 0.97 | 1.47 | 4.57 |  |  |
| **Pb** | **38.76** | 35.66 | 19.58 | 9.33 | 87.78 | 1.41±2 | 119.93 |
| **Sr** | **41.86** | 40.26 | 10.97 | 25.76 | 63.75 |  |  |
| **Zn** | **34.45** | 32.06 | 8.28 | 22.26 | 57.39 | 33±4 | 65.67 |

| **Descriptive statistics of stems of the *T. officinale*** | | | | | | | | | | | | | |
| --- | --- | --- | --- | --- | --- | --- | --- | --- | --- | --- | --- | --- | --- |
|  | **Mean** | **Med.** | **Min.** | **Max.** | **S.D.** | **C^1^** | **C^2^** | **C^3^** | **C^4^** | **C^5^** | **C^6^** | **C^7^** | **C^8^** |
| **Al** | 1340.9 | **1061** | 373.13 | 4719.61 | 876.29 |  |  |  |  |  | 65-2612 |  |  |
| **As** | 3.06 | **2.86** | 2.4 | 3.91 | 0.77 |  |  |  |  |  |  |  |  |
| **Ba** | 25.94 | **25.72** | 6.86 | 60.24 | 10.35 |  |  |  |  |  |  |  |  |
| **Cd** | 0.6 | **0.53** | 0.36 | 1.46 | 0.26 | 1.46±0.3 | 0.66 | 0.39 | 0.83-1.55 | 1.0-1.9 | 0.19-1.3 | 0.2-0.41 | 0.55-3.11 |
| **Co** | 1.85 | **1.6** | 0.91 | 7.85 | 1.31 |  |  |  |  |  |  | 0.39-0.61 |  |
| **Cr** | 9.43 | **5.47** | 2.55 | 41.58 | 8.5 | BDL | 3.16 |  |  |  | 0.5-2.20 | 3.9-4.7 | 4.53-61.72 |
| **Cu** | 13.41 | **11.45** | 7.29 | 50.38 | 7.45 | 9±1 | 52.67 | 9.2 | 8.0-21.8 | 10.1-13.9 | 4.0-22 | 11.8-14.3 | 2.1-58.41 |
| **Fe** | 1390.11 | **1184.35** | 365.29 | 5123.55 | 855.92 | 470±24 | 666.67 | 126 | 235-1056 |  |  |  | 80-3916 |
| **Mn** | 59.06 | **53.79** | 29.03 | 147.46 | 22.18 |  |  | 43 | 19-58 |  | 4.4-33 |  | 11.82-276.95 |
| **Mo** | 3.39 | **3.1** | 2.08 | 6.96 | 1.18 |  |  |  |  |  |  |  |  |
| **Ni** | 4.58 | **3.89** | 1.79 | 19.49 | 3.31 |  |  | 0.9 | 1.8-3.2 | 2.9-6.1 |  | 2.2-2.8 | 2.38-22.69 |
| **Pb** | 122.91 | **109.57** | 24.28 | 476.97 | 89.25 | 3.32±1 | 153.33 |  | 6.6-13 | 4.0-24 | 0.8-5.2 | 3.1-3.3 | 0.5-45 |
| **Sr** | 35.99 | **33.93** | 22.49 | 63.12 | 9.08 |  |  |  |  |  |  | 43.5-44.9 |  |
| **Zn** | 70.66 | **71.85** | 31.41 | 216.58 | 32.24 | 44±9 | 135 | 34 | 44-203 | 27.4-86 | 17-90 | 45-175.6 | 29-261.4 |

C^1^: Bini et al. 2012’s control sample results, C^2^: Bini et al. 2012’s examined sample results, C^3^: Czarnowska et al. 2000’s control sample results, C^4^: Czarnowska et al. 2000’s examined sample results, C^5^: Diatta et al. 2003’s examined sample results, C^6^: Djiingova and Kuleff, 1999’s examined sample results, C^7^: Gworek et al. 2011’s examined sample results, C^8^: Keane et al. 2001’s examined sample results

| **Descriptive statistics of flower of the *T. officinale* (bold numbers represent accepted averages taking into account the distributions of the data-normal or log normal)** | | | | | |
| --- | --- | --- | --- | --- | --- |
|  | Mean | Med. | Min. | Max. | S.D. |
| **Al** | 497.8125 | **445** | 142.00 | 1267.00 | 277.16 |
| **As** | **2.48** | 2.42 | 1.87 | 2.97 | 0.34 |
| **Ba** | **9.93** | 8.95 | 3.71 | 22.10 | 4.34 |
| **Cd** | **0.60** | 0.60 | 0.52 | 0.73 | 0.063 |
| **Co** | **1.40** | 1.03 | 0.59 | 1.89 | 0.35 |
| **Cr** | 4.17 | **3.34** | 1.81 | 14.60 | 2.50 |
| **Cu** | **12.16** | 11.65 | 8.62 | 16.80 | 2.14 |
| **Fe** | 544.13 | **509.50** | 188.00 | 1314.00 | 236.84 |
| **Mn** | **25.08** | 24.25 | 14.90 | 41.50 | 6.45 |
| **Mo** | **2.40** | 2.38 | 1.92 | 3.22 | 0.39 |
| **Ni** | 2.73 | **2.60** | 1.51 | 6.40 | 0.98 |
| **Pb** | 45.55 | **38.10** | 11.60 | 165.00 | 32.03 |
| **Sr** | **10.12** | 9.59 | 5.26 | 16.50 | 2.98 |
| **Zn** | 42.94 | **39.85** | 25.80 | 97.50 | 13.72 |

Table S3 Kolmogorov-Smirnov and Shapiro-Wilk normality tests of elemental contents in *Taraxacum officinale* roots, stems and flowers

|  | Kolmogorov-Smirnov^a^ | | | Shapiro-Wilk | | |
| --- | --- | --- | --- | --- | --- | --- |
| Root | Statistic | df | Sig. | Statistic | df | Sig. |
| Al | 0.134 | 28 | **0.200**^*^ | 0.927 | 28 | **0.051** |
| As | 0.187 | 19 | **0.080** | 0.931 | 19 | **0.182** |
| Ba | 0.126 | 28 | **0.200**^*^ | 0.951 | 28 | **0.209** |
| Cd | 0.287 | 3 |  | 0.929 | 3 | 0.485 |
| Co | 0.254 | 26 | 0.000 | 0.703 | 26 | 0.000 |
| Cr | 0.149 | 28 | **0.112** | 0.853 | 28 | 0.001 |
| Cu | 0.139 | 28 | **0.177** | 0.961 | 28 | **0.377** |
| Fe | 0.112 | 28 | **0.200**^*^ | 0.964 | 28 | **0.423** |
| Mn | 0.104 | 28 | **0.200**^*^ | 0.953 | 28 | **0.235** |
| Mo | 0.317 | 22 | 0.000 | 0.729 | 22 | 0.000 |
| Ni | 0.203 | 18 | **0.048** | 0.874 | 18 | 0.020 |
| Pb | 0.143 | 28 | **0.149** | 0.932 | 28 | **0.068** |
| Sr | 0.163 | 28 | **0.056** | 0.927 | 28 | **0.053** |
| Zn | 0.139 | 28 | **0.174** | 0.939 | 28 | **0.105** |
| Stems | Kolmogorov-Smirnov^a^ | | | Shapiro-Wilk | | |
| Al | 0.174 | 33 | .012 | 0.808 | 33 | 0.000 |
| As | 0.267 | 3 |  | 0.952 | 3 | 0.576 |
| Ba | 0.173 | 33 | 0.014 | 0.886 | 33 | 0.002 |
| Cd | 0.333 | 14 | 0.000 | 0.596 | 14 | 0.000 |
| Co | 0.368 | 32 | 0.000 | 0.488 | 32 | 0.000 |
| Cr | 0.233 | 33 | 0.000 | 0.742 | 33 | 0.000 |
| Cu | 0.259 | 33 | 0.000 | 0.586 | 33 | 0.000 |
| Fe | 0.179 | 33 | 0.009 | 0.748 | 33 | 0.000 |
| Mn | 0.196 | 33 | 0.002 | 0.809 | 33 | 0.000 |
| Mo | 0.221 | 16 | 0.036 | 0.823 | 16 | 0.006 |
| Ni | 0.227 | 30 | 0.000 | 0.647 | 30 | 0.000 |
| Pb | 0.165 | 33 | 0.022 | 0.819 | 33 | 0.000 |
| Sr | 0.151 | 33 | **0.053** | 0.925 | 33 | 0.025 |
| Zn | 0.197 | 33 | 0.002 | 0.728 | 33 | 0.000 |
| Flowers | Kolmogorov-Smirnov^a^ | | | Shapiro-Wilk | | |
| Al | 0.170 | 32 | 0.019 | 0.874 | 32 | 0.001 |
| As | 0.147 | 11 | **0.200^*^** | 0.960 | 11 | **0.774** |
| Ba | 0.145 | 32 | **0.087** | 0.940 | 32 | **0.077** |
| Cd | 0.158 | 18 | **0.200^*^** | 0.900 | 18 | **0.056** |
| Co | 0.097 | 29 | **0.200^*^** | 0.939 | 29 | **0.096** |
| Cr | 0.209 | 32 | 0.001 | 0.744 | 32 | 0.000 |
| Cu | 0.147 | 32 | **0.075** | 0.966 | 32 | **0.393** |
| Fe | 0.135 | 32 | 0.146 | 0.921 | 32 | 0.022 |
| Mn | 0.115 | 32 | **0.200^*^** | 0.940 | 32 | **0.074** |
| Mo | 0.138 | 23 | **0.200^*^** | 0.919 | 23 | **0.064** |
| Ni | 0.155 | 24 | 0.142 | 0.793 | 24 | 0.000 |
| Pb | 0.218 | 31 | 0.001 | 0.818 | 31 | 0.000 |
| Sr | 0.100 | 32 | **0.200^*^** | 0.962 | 32 | **0.319** |
| Zn | 0.194 | 32 | 0.003 | 0.809 | 32 | 0.000 |
| a. Lilliefors Significance Correction | | | | | | |
| *. This is a lower bound of the true significance. | | | | | | |

Table S4 Relationship of elements in different parts of the *Taraxacum officinale* with each other (Spearman Correlation Coefficients)

| Spearman's rho | | Al | As | Ba | Cd | Co | Cr | Cu | Fe | Mn | Mo | Ni | Pb | Sr | Zn |
| --- | --- | --- | --- | --- | --- | --- | --- | --- | --- | --- | --- | --- | --- | --- | --- |
| **Flowers** | Al | **1.00** |  |  |  |  |  |  |  |  |  |  |  |  |  |
|  | As | -0.31 | **1.00** |  |  |  |  |  |  |  |  |  |  |  |  |
|  | Ba | **0.84^**^** | -0.27 | **1.00** |  |  |  |  |  |  |  |  |  |  |  |
|  | Cd | 0.12 | 0.47 | 0.11 | **1.00** |  |  |  |  |  |  |  |  |  |  |
|  | Co | 0.31 | -0.59 | 0.07 | 0.11 | **1.00** |  |  |  |  |  |  |  |  |  |
|  | Cr | **0.58^**^** | -0.50 | **0.60^**^** | 0.13 | 0.18 | **1.00** |  |  |  |  |  |  |  |  |
|  | Cu | **0.56^**^** | -0.28 | **0.62^**^** | -0.07 | 0.19 | 0.36^*^ | **1.00** |  |  |  |  |  |  |  |
|  | Fe | **0.93^**^** | -0.24 | **0.81^**^** | 0.12 | 0.21 | 0.55^**^ | **0.63^**^** | **1.00** |  |  |  |  |  |  |
|  | Mn | **0.86^**^** | **-0.49** | **0.82^**^** | 0.12 | 0.20 | 0.65^**^ | **0.57^**^** | **0.85^**^** | **1.00** |  |  |  |  |  |
|  | Mo | 0.21 | 0.12 | 0.14 | **0.71^**^** | 0.10 | 0.31 | -0.03 | 0.05 | 0.18 | **1.00** |  |  |  |  |
|  | Ni | 0.35 | -0.03 | 0.34 | 0.11 | 0.05 | 0.47^*^ | 0.30 | 0.42^*^ | 0.36 | 0.11 | **1.00** |  |  |  |
|  | Pb | **0.51^**^** | -0.22 | **0.60^**^** | -0.02 | 0.29 | 0.36^*^ | **0.68**^**^ | **0.58^**^** | 0.37^*^ | 0.10 | 0.05 | **1.00** |  |  |
|  | Sr | **0.64^**^** | -0.29 | **0.71^**^** | -0.04 | 0.24 | **0.51^**^** | **0.65**^**^ | **0.72^**^** | 0.48^**^ | 0.00 | 0.26 | **0.80^**^** | **1.00** |  |
|  | Zn | **0.50^**^** | -0.25 | **0.60^**^** | -0.04 | 0.16 | 0.32 | **0.72**^**^ | **0.57^**^** | 0.41^*^ | 0.03 | 0.04 | **0.82^**^** | **0.69^**^** | **1.00** |
|  | | **Al** | **As** | **Ba** | **Cd** | **Co** | **Cr** | **Cu** | **Fe** | **Mn** | **Mo** | **Ni** | **Pb** | **Sr** | **Zn** |
| **Stem**  **Root** | Al | **1.00** | 0.50 | **0.79^**^** | 0.18 | **0.84^**^** | **0.71^**^** | **0.66^**^** | **0.94^**^** | **0.86^**^** | 0.29 | **0.56^**^** | **0.57^**^** | 0.401^*^ | 0.409^*^ |
|  | As | 0.49^*^ | **1.00** | 0.50 |  | 0.50 | **0.50** | **0.50** | **0.50** | 0.50 | -0.50 | **0.50** | **0.50** | 0.50 | 0.50 |
|  | Ba | **0.64^**^** | 0.04 | **1.00** | 0.33 | **0.69^**^** | **0.74^**^** | **0.66^**^** | **0.80^**^** | **0.69^**^** | 0.29 | **0.67^**^** | **0.68^**^** | **0.66^**^** | **0.54^**^** |
|  | Cd | **-0.50** | 1.00^**^ | -1.00^**^ | **1.00** | 0.39 | **0.55^*^** | 0.20 | 0.22 | 0.23 | 0.26 | 0.20 | 0.38 | **0.57^*^** | 0.45 |
|  | Co | **0.60^**^** | 0.15 | **0.58^**^** | **1.00^**^** | **1.00** | **0.61^**^** | **0.58^**^** | **0.79^**^** | **0.74^**^** | 0.19 | **0.62^**^** | **0.63^**^** | 0.443^*^ | 0.47^**^ |
|  | Cr | 0.18 | 0.04 | 0.15 | **0.50** | 0.41^*^ | **1.00** | **0.56^**^** | **0.69^**^** | **0.50^**^** | 0.35 | **0.78^**^** | **0.62^**^** | **0.59^**^** | **0.56^**^** |
|  | Cu | **0.57^**^** | -0.07 | **0.62^**^** | **-0.50** | **0.53^**^** | -0.12 | **1.00** | **0.62^**^** | **0.52^**^** | 0.33 | 0.39^*^ | **0.86^**^** | 0.60^**^ | **0.59^**^** |
|  | Fe | **0.95^**^** | 0.49^*^ | **0.63^**^** | **1.00^**^** | **0.66^**^** | 0.16 | **0.57^**^** | **1.00** | **0.90^**^** | 0.23 | **0.51^**^** | **0.56^**^** | 0.45^**^ | 0.37^*^ |
|  | Mn | **0.82^**^** | 0.35 | **0.75^**^** | **-1.0^**^** | **0.58^**^** | 0.35 | 0.47^*^ | **0.83^**^** | **1.00** | 0.27 | 0.36 | 0.440^*^ | 0.373^*^ | 0.19 |
|  | Mo | -0.10 | 0.21 | -0.01 | -0.50 | -0.07 | 0.14 | -0.28 | -0.15 | 0.04 | **1.00** | -0.011 | 0.330 | 0.210 | 0.124 |
|  | Ni | 0.24 | 0.02 | 0.29 | -0.50 | **0.49^*^** | **0.86^**^** | 0.15 | 0.27 | 0.26 | 0.37 | **1.00** | **0.54^**^** | 0.47^**^ | **0.66^**^** |
|  | Pb | **0.59^**^** | 0.16 | 0.28 | 0.50 | 0.43^*^ | 0.44^*^ | 0.40^*^ | **0.63^**^** | **0.56^**^** | **-0.49^*^** | 0.34 | **1.00** | **0.71^**^** | **0.73^**^** |
|  | Sr | 0.27 | -0.17 | 0.48^**^ | -0.50 | 0.46^*^ | 0.25 | 0.43^*^ | 0.24 | 0.39^*^ | 0.24 | **0.49^*^** | 0.071 | **1.00** | **0.70^**^** |
|  | Zn | 0.22 | 0.34 | 0.07 | -0.50 | 0.27 | 0.39^*^ | 0.03 | 0.22 | 0.28 | 0.31 | 0.35 | 0.080 | 0.47^*^ | **1.00** |
| *. Correlation is significant at the 0.05 level (2-tailed). | | | | | | | | | | | | | | | |
| **. Correlation is significant at the 0.01 level (2-tailed). | | | | | | | | | | | | | | | |

Table S5a KMO and Bartlett's Tests for plant flowers

| Kaiser-Meyer-Olkin Measure of Sampling Adequacy. | | 0.528 |
| --- | --- | --- |
| Bartlett's Test of Sphericity | Approx. Chi-Square | 195.620 |
|  | df | 78 |
|  | Sig. | 0.000 |

Table S5b Total Variance Explained table of elements in plant flowers

| Component | Initial Eigenvalues | | | Rotation Sums of Squared Loadings | | |
| --- | --- | --- | --- | --- | --- | --- |
|  | Total | % of Variance | Cumulative % | Total | % of Variance | Cumulative % |
| 1 | 8.171 | 62.851 | 62.851 | 6.027 | 46.360 | 46.360 |
| 2 | 2.014 | 15.490 | 78.341 | 3.050 | 23.463 | 69.822 |
| 3 | 1.025 | 7.884 | 86.225 | 2.132 | 16.403 | 86.225 |
| Extraction Method: Principal Component Analysis. | | | | | | |

Table S5c Rotated Component Matrix^a^

|  | Component | | |
| --- | --- | --- | --- |
|  | 1 | 2 | 3 |
| Al | 0.917 | 0.201 | 0.126 |
| Ba | 0.874 | 0.385 | 0.094 |
| Cd | 0.056 | 0.203 | 0.894 |
| Co | 0.737 | 0.463 | 0.165 |
| Cr | 0.950 | 0.204 | 0.045 |
| Cu | 0.343 | 0.754 | -0.163 |
| Fe | 0.846 | 0.423 | 0.033 |
| Mn | 0.934 | 0.174 | 0.122 |
| Mo | 0.064 | -0.008 | 0.940 |
| Ni | 0.757 | 0.314 | -0.008 |
| Pb | 0.336 | 0.811 | 0.387 |
| Sr | 0.690 | 0.641 | 0.025 |
| Zn | 0.319 | 0.787 | 0.449 |
| Extraction Method: Principal Component Analysis.  Rotation Method: Varimax with Kaiser Normalization. | | | |
| a. Rotation converged in 4 iterations. | | | |

Table S5d KMO measure of sampling adequacy and Bartlett's test of elements in dandelion stem

| Kaiser-Meyer-Olkin Measure of Sampling Adequacy. | | 0.789 |
| --- | --- | --- |
| Bartlett's Test of Sphericity | Approx. Chi-Square | 572.817 |
|  | df | 78 |
|  | Sig. | 0.000 |

Table S5e Total variance explained by dandelion stems

| Component | Initial Eigenvalues | | | Rotation Sums of Squared Loadings | | |
| --- | --- | --- | --- | --- | --- | --- |
|  | Total | % of Variance | Cumulative % | Total | % of Variance | Cumulative % |
| 1 | 10.004 | 76.952 | 76.952 | 8.756 | 67.352 | 67.352 |
| 2 | 1.865 | 14.350 | 91.302 | 3.113 | 23.949 | 91.302 |
| Extraction Method: Principal Component Analysis. | | | | | | |

Table S5f Rotated Component Matrix^a^ of dandelion stem

|  | Component | |
| --- | --- | --- |
|  | 1 | 2 |
| Al | 0.877 | 0.424 |
| Ba | 0.484 | 0.827 |
| Cd | 0.870 | -0.065 |
| Co | 0.896 | 0.345 |
| Cr | 0.944 | 0.180 |
| Cu | 0.939 | 0.149 |
| Fe | 0.894 | 0.420 |
| Mn | 0.834 | 0.505 |
| Mo | -0.147 | 0.879 |
| Ni | 0.956 | 0.215 |
| Pb | 0.952 | 0.176 |
| Sr | 0.422 | 0.866 |
| Zn | 0.951 | 0.200 |
| Extraction Method: Principal Component Analysis.   Rotation Method: Varimax with Kaiser Normalization. | | |
| a. Rotation converged in 3 iterations. | | |

Table S6 Kruskal-Wallis H (KW-H) test for dandelion

| *Test Statistics^a,b^* | | | | | | | | | | | | | | | |
| --- | --- | --- | --- | --- | --- | --- | --- | --- | --- | --- | --- | --- | --- | --- | --- |
|  | Al | | As | Ba | Cd | Co | Cr | Cu | Fe | Mn | Mo | Ni | Pb | Sr | Zn |
| KW-H | 36.289 | 2.197 | | 58.944 | 3.066 | 25.748 | 17.305 | 1.036 | 39.408 | 55.385 | 11.543 | 16.756 | 32.292 | 64.382 | 42.714 |
| df | 2 | 2 | | 2 | 2 | 2 | 2 | 2 | 2 | 2 | 2 | 2 | 2 | 2 | 2 |
| Asymp. Sig. | 0.000 | 0.333 | | 0.000 | 0.216 | 0.000 | 0.000 | 0.596 | 0.000 | 0.000 | 0.003 | 0.000 | 0.000 | 0.000 | 0.000 |
| a. Kruskal Wallis Test | | | | | | | | | | | | | | | |
| b. Grouping Variable: Plant parts | | | | | | | | | | | | | | | |

Table S7 Dandelion Tamhane T2 Multiple Comparisons test table

| Dependent Variable | | | Mean Difference (I-J) | Std. Error | Sig. | 95% Confidence Interval | |
| --- | --- | --- | --- | --- | --- | --- | --- |
|  |  |  |  |  |  | Lower Bound | Upper Bound |
| Al | Root | Stem | -212.94 | 187.86 | 0.60 | -675.34 | 249.46 |
|  |  | Flower | 630.19^*^ | 120.09 | 0.00 | 330.08 | 930.29 |
|  | Stem | Flower | 843.13^*^ | 160.22 | 0.00 | 443.20 | 1243.05 |
| As | Root | Stem | -0.37 | 0.46 | 0.87 | -3.31 | 2.56 |
|  |  | Flower | 0.20 | 0.16 | 0.50 | -0.20 | 0.60 |
|  | Stem | Flower | 0.58 | 0.46 | 0.69 | -2.45 | 3.60 |
| Ba | Root | Stem | 6.13 | 2.60 | 0.06 | -0.26 | 12.51 |
|  |  | Flower | 22.13^*^ | 2.02 | 0.00 | 17.06 | 27.20 |
|  | Stem | Flower | 16.01^*^ | 1.96 | 0.00 | 11.15 | 20.86 |
| Cd | Root | Stem | 0.01 | 0.10 | 1.00 | -0.30 | 0.31 |
|  |  | Flower | 0.01 | 0.07 | 1.00 | -0.47 | 0.48 |
|  | Stem | Flower | 0.00 | 0.07 | 1.00 | -0.20 | 0.19 |
| Co | Root | Stem | -0.40 | 0.28 | 0.41 | -1.09 | 0.29 |
|  |  | Flower | 0.41 | 0.17 | 0.06 | -0.02 | 0.84 |
|  | Stem | Flower | 0.81^*^ | 0.24 | 0.01 | 0.21 | 1.41 |
| Cr | Root | Stem | -4.94^*^ | 1.58 | 0.01 | -8.88 | -0.99 |
|  |  | Flower | 0.32 | 0.71 | 0.96 | -1.43 | 2.08 |
|  | Stem | Flower | 5.26^*^ | 1.55 | 0.01 | 1.40 | 9.12 |
| Cu | Root | Stem | 0.24 | 1.66 | 1.00 | -3.85 | 4.33 |
|  |  | Flower | 1.50 | 1.11 | 0.46 | -1.28 | 4.27 |
|  | Stem | Flower | 1.25 | 1.35 | 0.74 | -2.13 | 4.63 |
| Fe | Root | Stem | -235.44 | 176.42 | 0.46 | -670.47 | 199.59 |
|  |  | Flower | 610.55^*^ | 103.31 | 0.00 | 352.34 | 868.76 |
|  | Stem | Flower | 845.00^*^ | 154.78 | 0.00 | 458.94 | 1233.05 |
| Mn | Root | Stem | -18.90^*^ | 4.63 | 0.00 | -30.30 | -7.50 |
|  |  | Flower | 15.06^*^ | 2.81 | 0.00 | 8.02 | 22.09 |
|  | Stem | Flower | 33.96^*^ | 4.01 | 0.00 | 23.93 | 43.98 |
| Mo | Root | Stem | 0.09 | 0.54 | 1.00 | -1.26 | 1.44 |
|  |  | Flower | 1.08 | 0.46 | 0.08 | -0.10 | 2.26 |
|  | Stem | Flower | 0.99^*^ | 0.31 | 0.01 | 0.18 | 1.80 |
| Ni | Root | Stem | -2.056^*^ | 0.65 | 0.01 | -3.67 | -0.44 |
|  |  | Flower | -0.20 | 0.30 | 0.89 | -0.96 | 0.56 |
|  | Stem | Flower | 1.86^*^ | 0.64 | 0.02 | 0.26 | 3.45 |
| Pb | Root | Stem | -84.17^*^ | 15.97 | 0.00 | -124.19 | -44.15 |
|  |  | Flower | -6.80 | 6.84 | 0.69 | -23.69 | 10.10 |
|  | Stem | Flower | 77.38^*^ | 16.57 | 0.00 | 36.11 | 118.64 |
| Sr | Root | Stem | 5.88 | 2.61 | 0.08 | -0.55 | 12.31 |
|  |  | Flower | 31.74^*^ | 2.14 | 0.00 | 26.34 | 37.15 |
|  | Stem | Flower | 25.86^*^ | 1.67 | 0.00 | 21.71 | 30.02 |
| Zn | Root | Stem | -36.22^*^ | 5.84 | 0.00 | -50.82 | -21.62 |
|  |  | Flower | -8.49^*^ | 2.89 | 0.02 | -15.61 | -1.37 |
|  | Stem | Flower | 27.74^*^ | 6.12 | 0.00 | 12.53 | 42.94 |
| *. The mean difference is significant at the 0.05 level. | | | | | | | |

Table S8 Descriptive Statistics of SAF

|  | N | Minimum | Maximum | Mean | Std. Dev. | Skewness | | Kurtosis | |
| --- | --- | --- | --- | --- | --- | --- | --- | --- | --- |
|  | Statistic | Statistic | Statistic | Statistic | Statistic | Statistic | Std. Err. | Statistic | Std. Err. |
| SAF_Al | 33 | 0.00 | 0.06 | 0.02 | 0.01 | 2.42 | 0.41 | 7.96 | 0.80 |
| SAF_As | 3 | 0.19 | 0.43 | 0.27 | 0.14 | 1.72 | 1.22 |  |  |
| SAF_Ba | 33 | 0.01 | 0.16 | 0.04 | 0.03 | 2.21 | 0.41 | 6.20 | 0.80 |
| SAF_Cd | 0 |  |  |  |  |  |  |  |  |
| SAF_Co | 3 | 0.05 | 0.52 | 0.21 | 0.27 | 1.73 | 1.22 |  |  |
| SAF_Cr | 32 | 0.02 | 0.26 | 0.09 | 0.07 | 1.10 | 0.41 | 0.11 | 0.81 |
| SAF_Cu | 33 | 0.13 | 0.71 | 0.25 | 0.12 | 2.79 | 0.41 | 8.61 | 0.80 |
| SAF_Fe | 33 | 0.01 | 0.10 | 0.04 | 0.02 | 1.53 | 0.41 | 3.77 | 0.80 |
| SAF_Mn | 33 | 0.04 | 0.14 | 0.06 | 0.02 | 1.63 | 0.41 | 3.52 | 0.80 |
| SAF_Mo | 0 |  |  |  |  |  |  |  |  |
| SAF_Ni | 29 | 0.04 | 0.27 | 0.11 | 0.06 | 1.05 | 0.43 | 0.96 | 0.85 |
| SAF_Pb | 29 | 0.23 | 9.37 | **2.41** | 2.04 | 1.71 | 0.43 | 3.52 | 0.85 |
| SAF_Sr | 33 | 0.08 | 0.60 | 0.20 | 0.11 | 1.92 | 0.41 | 4.19 | 0.80 |
| SAF_Zn | 24 | 0.18 | 1.05 | 0.51 | 0.22 | 0.86 | 0.47 | 0.27 | 0.92 |

**Descriptive Statistics of RAF**

|  | N | Minimum | Maximum | Mean | Std. Dev. | Skewness | | Kurtosis | |
| --- | --- | --- | --- | --- | --- | --- | --- | --- | --- |
|  | Statistic | Statistic | Statistic | Statistic | Statistic | Statistic | Std. Err. | Statistic | Std. Err. |
| RAF_Al | 28 | 0.00 | 0.03 | 0.01 | 0.01 | 0.39 | 0.44 | 0.13 | 0.86 |
| RAF_As | 15 | 0.08 | 3.20 | 0.44 | 0.77 | 3.73 | 0.58 | 14.19 | 1.12 |
| RAF_Ba | 28 | 0.02 | 0.15 | 0.05 | 0.03 | 2.21 | 0.44 | 7.33 | 0.86 |
| RAF_Cd | 2 | 0.05 | 0.05 | 0.05 | 0.00 |  |  |  |  |
| RAF_Co | 3 | 0.04 | 0.33 | 0.15 | 0.16 | 1.66 | 1.22 |  |  |
| RAF_Cr | 27 | 0.01 | 0.12 | 0.04 | 0.03 | 1.69 | 0.45 | 2.50 | 0.87 |
| RAF_Cu | 28 | 0.12 | 0.49 | 0.26 | 0.10 | 0.51 | 0.44 | -0.28 | 0.86 |
| RAF_Fe | 28 | 0.01 | 0.07 | 0.03 | 0.01 | 1.12 | 0.44 | 3.90 | 0.86 |
| RAF_Mn | 28 | 0.02 | 0.09 | 0.04 | 0.02 | 0.74 | 0.44 | 0.81 | 0.86 |
| RAF_Mo | 0 |  |  |  |  |  |  |  |  |
| RAF_Ni | 17 | 0.02 | 0.14 | 0.06 | 0.03 | 1.09 | 0.55 | 2.85 | 1.06 |
| RAF_Pb | 24 | 0.07 | **2.32** | 0.93 | 0.62 | 0.87 | 0.47 | 0.20 | 0.92 |
| RAF_Sr | 28 | 0.09 | 0.66 | 0.24 | 0.14 | 1.75 | 0.44 | 3.12 | 0.86 |
| RAF_Zn | 19 | 0.11 | 0.59 | 0.27 | 0.13 | 1.19 | 0.52 | 1.45 | 1.01 |

**Descriptive Statistics of FAF**

|  | N | Minimum | Maximum | Mean | Std. Dev. | Skewness | | Kurtosis | |
| --- | --- | --- | --- | --- | --- | --- | --- | --- | --- |
|  | Statistic | Statistic | Statistic | Statistic | Statistic | Statistic | Std. Err. | Statistic | Std. Err. |
| FAF_Al | 32 | 0.001 | 0.014 | 0.006 | 0.00 | 1.07 | 0.41 | 0.99 | 0.81 |
| FAF_As | 9 | 0.06 | 1.19 | 0.32 | 0.34 | 2.50 | 0.72 | 6.77 | 1.40 |
| FAF_Ba | 32 | 0.00 | 0.04 | 0.02 | 0.01 | 0.90 | 0.41 | 0.00 | 0.81 |
| FAF_Cd | 5 | 0.04 | 0.27 | 0.12 | 0.09 | 1.26 | 0.91 | 1.62 | 2.00 |
| FAF_Co | 3 | 0.03 | 0.20 | 0.09 | 0.10 | 1.71 | 1.22 |  |  |
| FAF_Cr | 31 | 0.01 | 0.14 | 0.04 | 0.03 | 1.78 | 0.42 | 2.84 | 0.82 |
| FAF_Cu | 32 | 0.13 | 0.46 | 0.23 | 0.08 | 1.09 | 0.41 | 1.16 | 0.81 |
| FAF_Fe | 32 | 0.01 | 0.04 | 0.01 | 0.01 | 1.75 | 0.41 | 2.94 | 0.81 |
| FAF_Mn | 32 | 0.02 | 0.05 | 0.03 | 0.01 | 1.21 | 0.41 | 1.40 | 0.81 |
| FAF_Mo | 0 |  |  |  |  |  |  |  |  |
| FAF_Ni | 24 | 0.00 | 0.22 | 0.07 | 0.04 | 2.03 | 0.47 | 7.25 | 0.92 |
| FAF_Pb | 27 | 0.07 | 3.54 | **0.99** | 0.87 | 1.35 | 0.45 | 1.54 | 0.87 |
| FAF_Sr | 32 | 0.02 | 0.19 | 0.06 | 0.03 | 2.30 | 0.41 | 6.62 | 0.81 |
| FAF_Zn | 24 | 0.09 | 0.81 | 0.34 | 0.15 | 1.36 | 0.47 | 3.00 | 0.92 |

**Descriptive Statistics of TFs**

|  | N | Minimum | Maximum | Mean | Std. Deviation | Skewness | | Kurtosis | |
| --- | --- | --- | --- | --- | --- | --- | --- | --- | --- |
|  | Statistic | Statistic | Statistic | Statistic | Statistic | Statistic | Std. Error | Statistic | Std. Error |
| TFs_Al | 27 | 0.32 | 8.90 | **1.57** | 1.67 | 3.45 | 0.45 | 14.61 | 0.87 |
| TFs_As | 3 | 0.94 | 1.18 | **1.09** | 0.13 | -1.69 | 1.22 |  |  |
| TFs_Ba | 27 | 0.25 | 2.04 | 0.86 | 0.42 | 1.60 | 0.45 | 3.58 | 0.87 |
| TFs_Cd | 0 |  |  |  |  |  |  |  |  |
| TFs_Co | 24 | 0.38 | 3.92 | **1.38** | 0.70 | 2.07 | 0.47 | 6.82 | 0.92 |
| TFs_Cr | 27 | 0.40 | 18.66 | **3.36** | 4.02 | 2.54 | 0.45 | 7.69 | 0.87 |
| TFs_Cu | 27 | 0.48 | 2.31 | **1.08** | 0.49 | 1.23 | 0.45 | 1.04 | 0.87 |
| TFs_Fe | 27 | 0.37 | 7.05 | **1.46** | 1.31 | 3.22 | 0.45 | 12.87 | 0.87 |
| TFs_Mn | 27 | 0.66 | 3.83 | **1.61** | 0.73 | 1.22 | 0.45 | 2.19 | 0.87 |
| TFs_Mo | 13 | 0.30 | 2.51 | **1.11** | 0.71 | 0.98 | 0.62 | 0.01 | 1.19 |
| TFs_Ni | 16 | 0.00 | 5.77 | **1.91** | 1.54 | 1.24 | 0.56 | 1.10 | 1.09 |
| TFs_Pb | 27 | 0.49 | 10.65 | **3.80** | 2.76 | 0.96 | 0.45 | 0.23 | 0.87 |
| TFs_Sr | 27 | 0.48 | 1.40 | 0.91 | 0.24 | -0.18 | 0.45 | -0.32 | 0.87 |
| TFs_Zn | 27 | 0.96 | 5.47 | **2.19** | 1.02 | 1.39 | 0.45 | 2.65 | 0.87 |

**Descriptive Statistics of TFf**

|  | N | Minimum | Maximum | Mean | Std. Dev. | Skewness | | Kurtosis | |
| --- | --- | --- | --- | --- | --- | --- | --- | --- | --- |
|  | Statistic | Statistic | Statistic | Statistic | Statistic | Statistic | Std. Error | Statistic | Std. Error |
| TFf_Al | 27 | 0.16 | 2.32 | 0.55 | 0.50 | 2.53 | 0.45 | 6.60 | 0.87 |
| TFf_As | 8 | 0.55 | 1.29 | 0.97 | 0.26 | -0.27 | 0.75 | -1.12 | 1.48 |
| TFf_Ba | 27 | 0.10 | 0.75 | 0.33 | 0.17 | 1.12 | 0.45 | 1.07 | 0.87 |
| TFf_Cd | 2 | 0.84 | 1.30 | **1.07** | 0.33 |  |  |  |  |
| TFf_Co | 22 | 0.17 | 1.81 | 0.83 | 0.38 | 0.82 | 0.49 | 1.10 | 0.95 |
| TFf_Cr | 27 | 0.24 | 5.01 | **1.40** | 1.21 | 1.79 | 0.45 | 2.91 | 0.87 |
| TFf_Cu | 27 | 0.47 | 1.95 | **1.00** | 0.37 | 1.08 | 0.45 | 0.83 | 0.87 |
| TFf_Fe | 27 | 0.16 | 1.93 | 0.54 | 0.35 | 2.48 | 0.45 | 8.35 | 0.87 |
| TFf_Mn | 27 | 0.29 | 1.37 | 0.69 | 0.28 | 0.77 | 0.45 | -0.06 | 0.87 |
| TFf_Mo | 19 | 0.00 | 1.33 | 0.61 | 0.48 | -0.07 | 0.52 | -1.55 | 1.01 |
| TFf_Ni | 13 | 0.00 | 2.95 | 0.95 | 0.78 | 1.34 | 0.62 | 2.75 | 1.19 |
| TFf_Pb | 26 | 0.32 | 4.44 | **1.46** | 1.01 | 1.49 | 0.46 | 2.19 | 0.89 |
| TFf_Sr | 27 | 0.09 | 0.44 | 0.25 | 0.09 | 0.30 | 0.45 | 0.16 | 0.87 |
| TFf_Zn | 27 | 0.67 | 2.11 | **1.30** | 0.41 | 0.34 | 0.45 | -0.98 | 0.87 |

Table S9 Comparison of bioaccumulation and translocation factors of elements

|  | **SAF** | **RAF** | **FAF** | **TFs** | **TFf** |
| --- | --- | --- | --- | --- | --- |
| Al | 0.02 | 0.01 | 0.006 | **1.57** | 0.55 |
| As | 0.27 | 0.44 | 0.32 | **1.09** | 0.97 |
| Ba | 0.04 | 0.05 | 0.02 | 0.86 | 0.33 |
| Cd |  | 0.05 | 0.12 |  | **1.07** |
| Co | 0.21 | 0.15 | 0.09 | **1.38** | 0.83 |
| Cr | 0.09 | 0.04 | 0.04 | **3.36** | **1.4** |
| Cu | 0.25 | 0.26 | 0.23 | **1.08** | **1** |
| Fe | 0.04 | 0.03 | 0.01 | **1.46** | 0.54 |
| Mn | 0.06 | 0.04 | 0.03 | **1.61** | 0.69 |
| Mo |  |  |  | **1.11** | 0.61 |
| Ni | 0.11 | 0.06 | 0.07 | **1.91** | 0.95 |
| Pb | **2.41** | 0.93 | **0.99** | **3.8** | **1.46** |
| Sr | 0.2 | 0.24 | 0.06 | 0.91 | 0.25 |
| Zn | 0.51 | 0.27 | 0.34 | **2.19** | **1.3** |
